# System Integration - A Major Step toward Lab on a Chip

**DOI:** 10.1186/1754-1611-5-6

**Published:** 2011-05-25

**Authors:** Mandy LY Sin, Jian Gao, Joseph C Liao, Pak Kin Wong

**Affiliations:** 1 Department of Aerospace and Mechanical Engineering, University of Arizona, Tucson, AZ 85721, USA; 2 Department of Chemical Engineering, Shandong Polytechnic University, Jinan, 250353, China; 3 Department of Urology, Stanford University, 300 Pasteur Drive, S-287, Stanford, CA 94305, USA; 4 Biomedical Engineering and Bio5 Institute, University of Arizona, Tucson, AZ 85721, USA

## Abstract

Microfluidics holds great promise to revolutionize various areas of biological engineering, such as single cell analysis, environmental monitoring, regenerative medicine, and point-of-care diagnostics. Despite the fact that intensive efforts have been devoted into the field in the past decades, microfluidics has not yet been adopted widely. It is increasingly realized that an effective system integration strategy that is low cost and broadly applicable to various biological engineering situations is required to fully realize the potential of microfluidics. In this article, we review several promising system integration approaches for microfluidics and discuss their advantages, limitations, and applications. Future advancements of these microfluidic strategies will lead toward translational lab-on-a-chip systems for a wide spectrum of biological engineering applications.

## Background

Microfluidics is a multidisciplinary field investigating the behavior and the manipulation of small amounts of fluids with characteristic length scales from nanometers to hundreds of micrometers [1,2]. The field has been under intensive development for over 20 years as a result of the emergence of microelectromechanical systems. The dramatic change in the length scale offer many new techniques due to the unique importance of phenomena at the microscale such as the domination of surface forces over inertial forces, the laminar nature of fluid flow, fast thermal relaxation and length scale matching with the electric double layer [3]. From a technological point of view, microfluidics offers many advantages including low fluid volumes (less reagents and lower cost), short assay time, low power consumption, rapid generation of small liquid compartments and high degree of parallelization [4-11]. Despite the fact that the inherent advantages of microfluidics are highly promising for realizing the concept of lab-on-a-chip, microfluidics has not been widely adopted in biological engineering and medical applications. By now, the most successful portable bioanalytical platforms with the largest market share are test stripes, which were introduced in the middle of 1980s [12-14].

In the past decades, microfluidics has undergone rapid development with numerous new fabrication techniques and device designs. There are a large number of publications and patents of microfluidic devices functioning as pumps [12,13], mixers [14-16], concentrators [17], and valves [18-20], which are the building blocks for creating functional bioreactors and lab-on-a-chip systems. Nevertheless, a major hurdle for transforming microfluidics into practical applications is the integration of these components into a fully automated platform that can be conveniently accessed by the end users [21]. This is primarily due to the complexity of combining various components including bulky supporting equipments (e.g., pressure sources and cell culture modules), detection components (e.g., optics and engineering interfaces), and sample preparation modules (e.g., mixers and concentrators) into a single platform [22].

The major criteria for developing an integrated lab-on-a-chip system depend on the proposed applications and target markets of the products [23-39]. For example, it is widely believed that lab-on-a-chip technology will advance global health through the development of *in vitro *diagnostic devices for point-of-care testing (e.g., routine monitoring for chronic diseases and emergency testing for acute diseases) and advanced diagnostic devices in central laboratory testing [40-43]. In a central laboratory setting, sensitivity and specificity of the test are often the major considerations when supporting infrastructures are available and a high-cost, high-performance system is affordable. Due to the lack of sufficient trained personnel in remote locations (e.g., airports or train stations), diagnostic assays should allow automated operations by untrained personnel and the results should be easily interpreted by the end users. In resource-limited settings (e.g., a rural clinic), the cost, portability and shelf life represent the major constraints for the development of the system and the ability to transfer the test results to physicians in other locations for off-site diagnosis using the existing communication network is valuable [44]. The chip designers, therefore, should consider these issues and requirements according to the target applications at the preliminary stage.

In the past decades, numerous microfluidic techniques have been developed for a wide spectrum of biological engineering applications. These microfluidic systems have been successfully applied in laboratory scale applications [45]. However, most existing microfluidic systems are practically chip-in-a-lab, instead of lab-on-a-chip, and only possess limited functionalities [46]. Recently, several microfluidic strategies are emerging for effective integration of multiple microfluidic components towards fully automated lab-on-a-chip systems for sophisticated biomedical analyses [47]. In particular, capillary driven microfluidics, multilayer soft lithography, multiphase microfluidics, electrowetting-on-dielectric, electrokinetics, and centrifugal microfluidics are some of the most promising platforms for transforming microfluidics into various biological engineering applications. In this article, the basic principles, applications, strengths, and limitations of these microfluidic platforms are discussed.

## Capillary Driven and Paper-based Microfluidics

### Background

Test strips introduced in the middle 1980s are currently the most successful portable diagnostic platform commercially available. The major advantages of test strips include simplicity, portability and cost effectiveness [48]. In a test strip, the liquid transport is driven by capillary action of a fleece without the requirement of external transportation support. It offers single-use point-of-care diagnostics (qualitative or quantitative detection) such as cardiac marker assays and pregnancy test [49]. On the other hand, there is a rapid development of paper-based microfluidic devices, which are also driven by the capillary effect, through micropatterning of test paper with hydrophobic polymers for channeling the fluid into different regions [50]. Paper-based microfluidics has enhanced flexibility in the device design for versatile usage while the cost can be compatible to test strips.

### Technology

**Figure 1:**
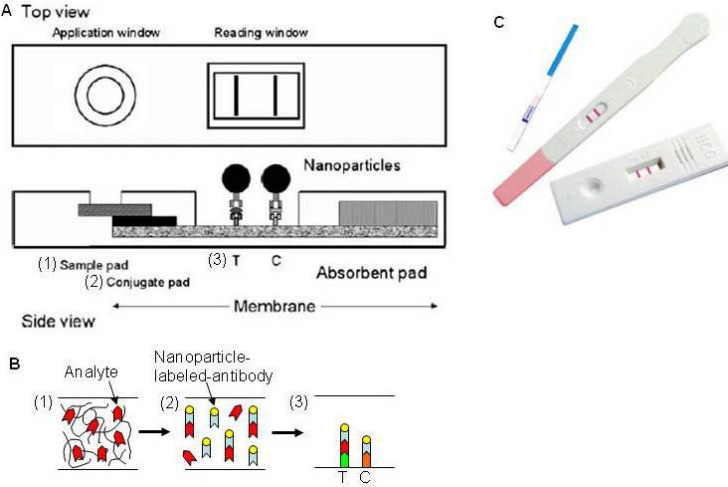
**Test strip platform**. (A) Schematic design of a test strip platform [51]. The sample fluid is drawn from the sample pad into the conjugate pad and membrane through the capillary action. (B) Immunoassays on the test strip. At the conjugate pad, nanoparticle-labeled-antibody rehydrate and bind with the specific antigen in the sample. Two different capturing antibodies are sprayed at the test line T and the control line C. At the test line, the antibody bind with the nanoparticle-labeled-antibody/antigen complex (positive assay) while the control line attach with the nanoparticle-labeled-antibody complex (proof of successful assay). (C) Image of commercially available pregnancy test strips [219].

The test stripe platform consists of fleeces, which can draw liquid through stripes using the capillary effect (Figure 1a). The sample liquid, such as urine and blood, reacts with the reactants pre-immobilized on the stripe. The capillary filling action can be influenced by the permeability, the roughness, the dimension, the surface properties, and the total number of capillaries inside the stripe [51]. The fleece can also serve as a sample filter, which is essential for processing many physiological and environmental samples. For example, in blood analysis, blood cells can be blocked from entering into the reaction chamber. This eliminates the need for centrifugal separation [52]. Adequate and precise incubation of the sample with the reactant is also required for reactions to occur. Incubation time can be controlled by slowing down the capillary flow with local modifications of the channel geometry and property [51]. It should be noted that metering of sample liquid in the test stripe is important for quantitative assay. To ensure the well defined amount of liquid has passed the detection zone, the start reservoir should be filled with enough sample liquid. The liquid flow stops automatically in the end reservoir when the whole piece of fleece is fully wetted with liquid. The detectable signal of the test stripe assay can be measured quantitatively by engineering interfaces or qualitatively by manual observation in the detection zone. For optical detection, the diagnostic section can be illuminated by a laser diode and the resulting fluorescence emission of the fluorescently labeled analytes can be detected by a photodiode [53,54]. For qualitative readout, the analytes can be bound to small gold nano particles or colored latex particles. Accumulation of the analytes at the detection zone can produce a readable signal [51] (Figure 1b). Bioanalytical assays can also be performed based on enzymatic reactions [55]. For instance, the amperometric signal generated by an enzymatic oxidation reaction, which depends on the concentration of the analyte, can be measured using an electronic interface.

**Figure 2:**
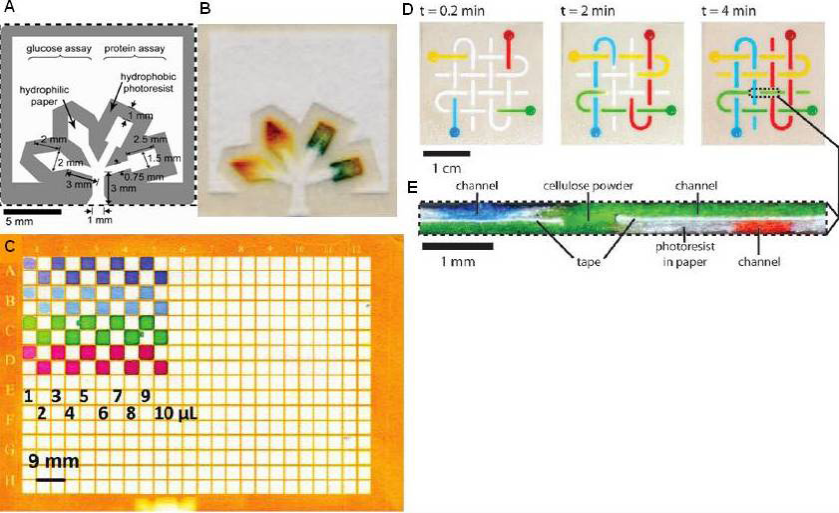
**Microfluidics paper based platform**. (A) Schematics of a microfluidic paper-based analytical device which can detect glucose and protein in urine simultaneously [71]. (B) Urine sample in a paper-based microfluidic device fabricated by photolithography [71]. (C) Image of a 384-zone paper plate produced with photolithography after applying a range of volumes (1-10 μL) of solutions of different dyes [60]. It shows the fluid isolation abilities of the zones although two zones in the fifth and sixth rows show small breaches in the hydrophobic walls. (D) A 3D microfluidic paper-based analytical device with four channels located at different plates without mixing their contents [56]. (E) Cross-section view of the device in (D) [56].

Paper-based microfluidics have been demonstrated in recent years [56]. These diagnostic devices are made of paper, which can act as the channel and physical filter for samples and reagents. The devices are usually fabricated by patterning a paper with spatial hydrophobic barriers such that the bounded regions become the hydrophilic channels (Figure 2). The channels can be either left open to the atmosphere or sealed to thin polymer sheets. The channel guides the fluids through capillary action. Similar to a test stripe, the capillary action can be controlled by the characteristics of the material and the environmental conditions (e.g., temperature and relative humidity). There are a number of methods for creating hydrophobic patterns, such as photolithography [57], plasma etching [58], and wax printing [59]. In these methods, the thickness of the paper determines the height of the channel while the patterning process defines the geometry of the channel. The fabrication process is followed by saturating the test zones with assay reagents using manual spotting or inkjet printing. The reagents in the device should be functional after they are fully dry. Most of the diagnostic analyses on paper-based microfluidics are based on colorimetric assays and the quantitative measurement can be achieved by reflectance detection [56]. In reflectance detection, the concentration of the analyte is related to the amount of light reflected from the surface of the test zone that can be captured by a desktop scanner or digital camera. Elaborate designs for more diverse applications have been created based on paper-based microfluidics. By patterning the paper into an array of circular test zones, such as 96-zone plates or 384-zone plates, high throughput assays can be accomplished providing an alternative to conventional microplates [60] (Figure 2d). Furthermore, 3D paper-based microfluidics have been developed by stacking layers of paper-based microfluidic devices with double-sided adhesive tape patterned with fluidic connections [61]. With the 3D networks of channels, multiple operational units can be combined into a single device [48] (Figure 2d and 2e). Existing microfluidic designs, such as the H-filter [62] and T-sensor [63], can be incorporated in the paper networks without pumping or pneumatic control systems [64].

### Applications

The test stripes have been the most widespread commercial platform for a large number of on-site diagnostic applications for over 20 years. The simplest example is pH measurement with the litmus paper [65]. More intricate structures such as fleece with multiple reactants and colorization or multiple fleeces with different zones of reactants can be applied in pregnancy tests, blood glucose monitoring, cardiovascular disease assays, and drug abuse tests. These detection platforms are already commercially distributed [66,67] (Figure 1c). For paper-based microfluidics, one of the first applications is urinalysis for glucose and bovine serum albumin [68]. Other bioassays include rapid blood typing [69] and salivary nitrite monitoring [70]. With 3D paper-based microfluidics, testing 4 different samples for 4 different analytes has been demonstrated on a single device [61].

### Strength and weakness of the platform

Capillary driven microfluidic platforms require only a simple actuation mechanism - capillary force with no pumping, valve or energy sources necessary. This makes system integration and on-site automated bioanalytical tests achievable. The devices are low-cost, small, and light weight making storage and transportation relatively easy. Furthermore, they can be disposed economically and safely by incineration. All these attractive features make capillary driven microfluidics a powerful platform for clinical diagnostics especially in resource-limited settings. However, the variations in viscosity and surface tension among different samples and changes in environmental conditions can significantly affect the wicking rate and the incubation time of the assays. Since active fluid control, e.g. mixing and concentration, is not available once the process starts, the efficiency could be a challenging issue for dilute samples. Furthermore, the shell-life of the reagents coated on the device surface, which could degrade over a certain period of time, can also influence the accuracy of the devices.

Paper-based microfluidics shares basic characteristics with test stripes. Although paper-based microfluidics is still in the developing stage, it shows a number of overwhelming characteristics over the conventional test strip. Firstly, multiplexing can be carried out simultaneously in different test zones on the paper without cross-contamination of the reagents. Secondly, on-site diagnostics for colorimetric assays can be easily achieved with cell phone cameras, scanners and other well-established communications infrastructure [71]. Last but not least, the design of 3D paper-based microfluidics can potentially implement more complex diagnostic assays while preserving its economical nature. However, paper-based microfluidics also shares similar technical issues with test stripes. In addition, there is one issue related to reflectance detection. The digital pictures (assay results) can vary based on the lighting conditions, the resolution of the camera, and the focus of the picture [56]. Color standards can be integrated in the device to eliminate this effect.

## Multilayer Soft Lithography

### Background

Multilayer soft lithography (MSL) applies pneumatic actuation for controlling large-scale microfluidic networks, which integrate thousands of fluidic components including mechanical valves, mixers, and pumps on a microfluidic chip [72]. MSL are fabricated by bonding multiple layers of elastomer, e.g., polydimethylsiloxane (PDMS), cast from micromachined molds [73]. PDMS based microfluidic devices have several advantages over silicon or glass based systems including its low cost, deformability, and optical transparency. PDMS elastomer also shows good biocompatibility, impermeability to water and permeability to gases. All these factors render PDMS an appropriate material implementing MSL with pneumatic fluid control for various biological engineering applications [74,75].

### Technology

**Figure 3:**
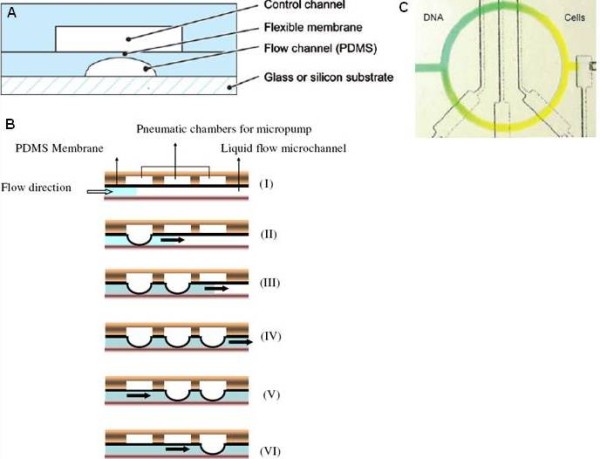
**Essential functional fluidic units on the multilayer soft lithography platform**. (A) A push-down valve [72]. (B) The cross-sectional view of the peristaltic pump [80]. (C) Peristaltic mixer [72].

The fundamental building block of the MSL platform is a pneumatic valve, which is made by sealing two layers of PDMS together (Figure 3a). The PDMS layers contain microchannels aligned perpendicularly for fluid transport and pneumatic control [72]. The valve is formed at the intersection of the pneumatic control channel and fluid transport channel. The operating mechanism of the microvalve relies on the high deformability of PDMS, which allows large actuation and blockage of the fluid transport channel with pneumatic control. Both push-up and push-down valves can be constructed by building the control channel layer below and above the fluid channel layer respectively. Volume containment ranging from pico- to nanoliters can be achieved with a pair of microvalves. Small dead volumes and relatively simple fabrication procedures have made these valves become one of the most prominent mechanical microvalves [76]. With a latching valve design, an on-chip demultiplexer has been demonstrated to reduce the number of off-chip controllers and only *n *pneumatic inputs are required to control *2*^*(n-1) *^independent latching valves [77].

The PDMS valve technology can also create other fundamental microfluidic components, such as peristaltic pumps and mixers. The pumping motion can be obtained by arranging an array of valves in series and actuating them in a peristaltic sequence (Figure 3b). Other designs of peristaltic micropumps have also been reported [78,79]. For instance, a pneumatic micropump with a serpentine-shape layout is capable of providing a broad pumping rate range from 0 to 539 μlh^-1 ^[80]. Besides pumping, mixing can also be accomplished by building a peristaltic pumping system with a channel of rotary geometry (Figure 3c). After two different reagents are injected into the rotary channel from the inlet and outlet valves respectively, both valves are closed and the peristaltic pump is activated. As the fluid flow inside the microchannel follows a hyperbolic velocity profile, the peristaltic pump driven motion leads to an increase in the interfacial area between the reagents and enhances the mixing. The rotary mixer has been utilized to perform reverse transcription polymerase chain reaction (PCR) and it has been demonstrated that the efficiency of gene expression analysis can be enhanced by 70% when compared to the diffusion limited case [81]. Beside the pneumatic control fluidic networks, other activation strategies for the multilayer soft lithography microfluidic systems can also be applied. For example, networks of fluidic gates controlled with a constant flow of Newtonian fluids in a three-layer PDMS structure have been demonstrated [82].

### Applications

Integrated microfluidic systems fabricated by MSL have demonstrated the capability to automate complex biological assay procedures and have been applied in various biomedical applications. Digital PCR for detecting copy number variations is one of the promising applications of the MSL microfluidic platform [83]. Accurate quantification of copy number variations for the human genome is essential for studying the association of such variations with human disorders [84]. However, conventional technologies, such as high density single nucleotide polymorphism microarrays and quantitative PCR, can at best distinguish a twofold difference in copy number variations. Digital PCR using a digital array can identify and quantify individual DNA molecules based on the principle of partitioning and it can differentiate as little as 15% differences in gene copy number [85]. Another application of the MSL microfluidic platform is high throughput gene expression analysis and over two thousand real-time PCR gene expression measurements in a single chip have been demonstrated [86]. The MSL microfluidic system is also capable of quantitative measurements with single-cell resolution [87]. Other applications of MSL microfluidics include a fully automated cell culture system, which can create culture media formulations in 96 independent culture chambers and maintain cell viability for weeks [88,89]. Furthermore, immunoassay based on MSL microfluidics has permitted multiplexed protein measurements using nanoliter-scale samples [90].

### Strength and weakness of the platform

MSL microfluidics using PDMS is a low-cost, robust, and easily configurable technology. A large number of microfluidic components, such as mixers, pumps and valves, can be integrated in a single chip allowing automation of complex biological analysis procedures. The high-throughput capacity of MSL microfluidics enables effective genetic analyses that are otherwise labor intensive and cost restrictive. Also, as the pneumatic valves are miniature, a single fluidic circuit can accommodate thousands of reaction chambers. Additional capacity can be achieved simply by including additional fluidic circuits, which makes the platform modular and applicable to various biological applications. Although MSL related applications, such as high-throughput sequencing technologies, provide a broad impact on the biomedical research and show many potential applications in clinical diagnostics, most of current MSL microfluidic systems are designed for laboratory scale research and require additional supporting equipment for the operation of the device. The current technology is difficult to be implemented as a portable bioanalytical device for field applications [91]. To implement the pneumatic controlled microfluidic network at the point of care necessary items such as external pressure sources, multiplexed gas valves, and detection modules are required to be integrated into the final system, which could present a technological hurdle for system integration [78,92].

## Multiphase Microfluidics

### Background

Multiphase microfluidics is a promising microfluidic technology with precise control of the fluid in the form of droplets. The essence of this technology is to generate a large number of droplets with uniform size and shape as independent bioreactors that can be transported, mixed, split, recombined and analyzed [93]. By performing the desired biochemical reactions in droplets, not only can the amount of reagents be reduced down to femtoliter volumes, but also the high surface-to-volume ratio of droplets can enhance the mass and heat transfer, which in turn accelerates the reaction. Furthermore, multiphase microfluidics is a unique technique that is capable of performing a large number of parallel experiments without significantly increasing the scale and complexity of the system.

### Technology

**Figure 4:**
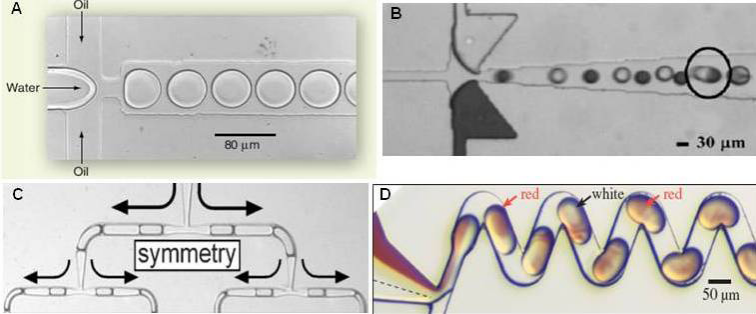
**Multiphase flow droplet microfluidics platform**. (A) Droplet formation in microchannels when two immiscible fluid (water and oil) streams merge [220]. (B) Fusion of alternately generated droplets in an expanding channel [99]. (C) Splitting of droplets at the T-junctions [102]. (D) Internal mixing within droplet through a winding microchannel [221]. Arrows show 'flipping' of coloured solution within the droplet.

Multiphase microfluidic systems are typically driven by external pressure sources. Droplets, or bioreacters, in sub-nanoliter volume can be formed spontaneously in the microchannel when two immiscible fluid streams such as water and oil merge [94] (Figure 4a). The droplets can be generated using two channel geometries: T-junction and flow-focusing. The T-junction droplet generator relies on the shear force created at the junction [95] whereas the flow-focusing droplet generator combines sheath flow with a restriction to generate droplets continuously [96]. The size of the droplets can be regulated by the channel geometry, fluid flow rates, and the relative viscosity between the two solutions [97,98]. With the flow-focusing structure, monodispersed pico- to femtoliter sized droplets can be generated at adjustable rates. The formation of more complex double emulsions, such as water-in-oil-in-water (W/O/W) and oil-in-water-in-oil (O/W/O), can be obtained using two consecutive flow-focusing devices [99,100]. Various droplet processes such as fusion, fission, and mixing have been demonstrated by adjusting the flow rate and channel designs [93]. For example, droplet fusion can be initiated by incorporating an expanded portion in the microchannel [101] (Figure 4b) while splitting droplets can utilize shear forces generated by appropriate channel designs, such as T-junctions [102] and branching channels [103] (Figure 4c). For droplets containing multiple reagents, mixing within a droplet can be enhanced geometrically using channels with bends and turns [104] or small protrusions [105], which create chaotic advection for folding and stretching of droplet contents (Figure 4d). Droplet incubation is another essential operation for various biochemical reactions. If the incubation time is long (e.g. over one hour), the droplets can be incubated in on-chip or off-chip reservoirs and reinjected into the device for further analysis. For short incubation time below one hour, a delay-line with a two-depth channel has been reported with minimum back pressure and low dispersion in incubation time [106]. Moreover, bubbles travelling in a microchannel can implement computation and represent a bit for transporting materials and performing logical control operations [107].

### Applications

Emulsions of aqueous droplets in oil have been used widely as microreactors for various biomedical applications as most of the biological reagents involved are in aqueous form. A number of approaches have been developed for merging different reagents into a common droplet for reaction confinement. For example, with alternating microdroplet generation followed by droplet fusion, the synthesis of semiconductor nanoparticles has been realized [99]. Recently, bacteria identification and detection of their susceptibility to antibiotics [108], single cell genetic analysis [109], and PCR for targeted sequencing [110,111] have been demonstrated by taking advantage of the ability to generate a large amount of droplet microreactors. Additional electrical components have also been integrated with the pressure driven platform for more versatile applications [110,111]. For example, dielectrophoresis has been employed for sorting different types of cells at rates up to 2000 droplets per second [112,113].

### Strength and weakness of the platform

The major advantages of multiphase microfluidics include the abilities to rapidly create a large number of reaction chambers and to precisely control the chemical reactions. These properties are particularly useful for high throughput analyses, such as single cell and single molecule studies. Since biochemical reactions are generally carried out in emulsions of aqueous droplets in oil, the droplets can isolate the reagents for avoiding cross contamination, evaporation of solvents, and unnecessary adsorption on the channel surface. In addition, the device involves no moving micromechanical structures. Therefore, the fabrication process for multiphase microfluidics is relatively simple and cost-effective. For applications that require precise manipulation of the droplets, controls over the surface properties of the channel are necessary because the wetting property of the fluid with respect to the channel wall is important in determining the droplet generation. Furthermore, the microfluidic operation of multiphase microfluidics is typically defined by the channel design and the device is not reconfigurable for other applications. Similar to other pressure driven microfluidic systems, the multiphase microfluidic platform requires supporting equipment, such as syringe pumps and control valves, and is difficult to implement at the point of care.

## EWOD Driven Droplet Microfluidics

### Background

Another droplet based microfluidic strategy is electrowetting-on-dielectric (EWOD) [114]. While droplet manipulations are involved, EWOD and multiphase microfluidics have different actuation mechanisms. In EWOD based microfluidics, droplet manipulation is achieved by electrowetting on an electrode array and does not require bulky equipment, such as a syringe pump. Various microfluidic operations, such as droplet creation, mixing, merging and splitting, can be performed by programming the applied voltage in the electrode array. Compared to other techniques, the EWOD driven droplet microfluidics platform is highly reconfigurable and requires only electronic interfaces to support its operation. These characteristics are ideal for bioanalytical processes that require complicated procedures and point-of-care diagnostics.

### Technology

**Figure 5:**
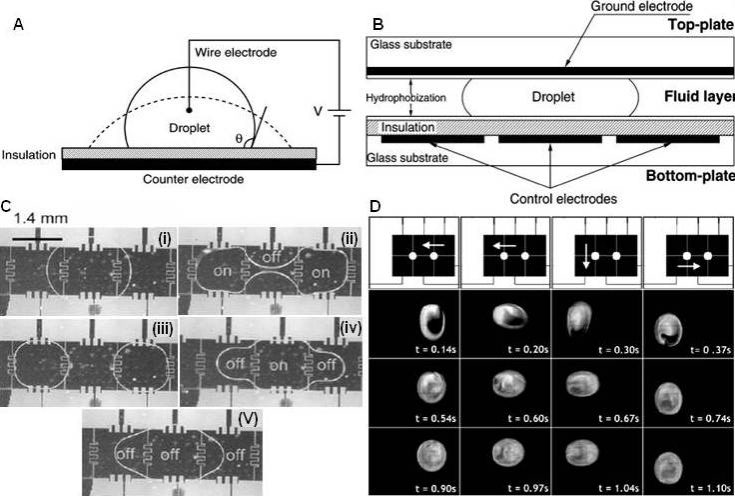
**EWOD driven droplet microfluidics platform**. (A) The electrowetting effect: the change of the wetting properties of a hydrophobic surface with an application of a voltage V between the droplet and a counter-electrode [222]. (B) Cross-sectional schematic diagram of the two plane EWOD driving platform [222]. (C) Consecutive images of droplet splitting and droplet fusion by turning on and off different electrodes [117]. (D) Mixing within droplet by maneuvering it in an irreversible electrode pattern [126]. Top row shows the pivot point about which the droplet moves in a 2 × 3 mixer. Sequential images of droplet mixing at 16 Hz (2-4 row).

EWOD is based on the electrowetting effect, which is the change of the surface energy of a surface as a result of an applied electric field [115] (Figure 5a). By applying an external electric field, the surface hydrophobicity decreases and in turn reduces the contact angle of the fluid. EWOD devices can be fabricated as a one or two plane system [116]. In the two plane system, the droplet is sandwiched between the electrodes covered with dielectric layers (Figure 5b). These dielectric coatings are hydrophobic and insulating in nature for providing a large contact angle and avoiding electrolysis. The top layer is often the ground electrode while the bottom layer is an array of electrodes to control droplet operations. In the one plane system, the droplet can be grounded from below using thin conductive lines on top of the insulating dielectric layer. The two plane device has less evaporation and greater exchange surface with the electrode due to the squeezing of the droplets in between two planes while the one plane device permits faster droplet manipulation and direct access to other lab automation equipment, such as liquid handlers, Surface Plasmon Resonance setups and Fourier Transform Infrared Reflectometry systems. Droplet creation can be accomplished from an on-chip reservoir by three steps. Firstly, activation of a series of electrodes adjacent to the reservoir initiates a liquid column to extrude from the reservoir. When the liquid column covers the electrode on which the droplet is to be formed, all the other electrodes are switched off to form a neck in the column. Then activation of the electrode inside the reservoir pulls back the liquid and breaks the neck to form a droplet [117]. With this method, nanoliter droplets could be generated with a standard deviation below 3% [118]. The size of the droplet can be controlled by the amplitude and frequency of the applied electric field, for example, higher frequency can produce smaller droplet. As uniform droplet dispensing is critical for performing assays on the EWOD platform, additional electronic modules have been developed for obtaining real-time feedback control of droplet generation [119]. Droplet maneuvering can be performed when electrical potentials are individually applied to an array of electrodes. The imbalance of the surface energy induces a net force on the fluid droplet and generates the droplet motion [120,121].

Other droplet-based operations, such as merging and mixing, can also be performed by programming the electric actuation signal [117,122]. For example, when two droplets are in close contact with a pair of electrodes, an applied potential of AC or pulsed DC will result in droplet coalescence within 100 μs [123,124] (Figure 5c). Mixing is a crucial step following droplet merging. The basic mechanism of mixing is the oscillation of a droplet between two electrodes so that advection inside the droplet is enhanced [125,126]. However, flow reversibility has been observed with a linear array of electrodes which limits the mixing efficiency. This can be improved by moving the droplet along an irreversible pattern that breaks the symmetry of the two inner circulating flows [127] (Figure 5d). Also, capacitance measurements have been used to determine the state of droplet mixing [128]. Accompanied with other electrokinetic forces, additional fluidic operations including separation and concentration can also be achieved [129,130]. Separation can be implemented by incorporating electrophoresis or dielectrophoresis within a droplet in which two different types of particles can be isolated into two regions inside a mother droplet followed by splitting the droplet. Concentration can be achieved using a similar procedure for droplets containing only one type of particle. On the other hand, optoelectrowetting on open, featureless, and photoconductive surfaces has also been reported for droplet manipulation [131-134]. This approach uses reconfigurable, low intensity optical patterns from a LCD display or a portable cellular phone to control droplet operations including transport, splitting, merging, and mixing.

### Applications

The EWOD driven microfluidic platform has been applied in various biological engineering applications. For instance, mass spectrometry, which is an important technique for proteomics, requires tedious processing steps including reduction, alkylation, and enzymatic digestion. The lack of a standard sample handling and processing platform has become one of the foremost limitations and EWOD is a powerful tool for performing automated proteomic sample processing [135,136]. On the other hand, the EWOD platform can not only perform complete mammalian cell culture [137], but also allowe cell isolation and single cell analysis [138]. Other application of EWOD includes multiplexed real-time PCR which has exhibited a high amplification efficiency [139].

### Strength and weakness of the platform

The EWOD driven microfluidic platform requires relatively simple microfabrication procedures as it involves no moveable mechanical parts in the device. Furthermore, bulky instruments are not required for EWOD operations. Microfluidic operations, such as mixing, merging, sorting, and separation, can be performed on the same set of electrodes by proper programming of the actuation signal. These render EWOD a promising platform for performing complicated bioanalytical procedures and potentially biomedical analyses in resource limited settings. However, addressing a large array of microelectrodes independently is required for high-throughput applications. To this end, multilayer printed circuit boards which allow isolation of electrical wires in different layers can be applied to control a large number of electrodes individually [140]. Another promising solution is optoelectrowetting, which provides a highly flexible interface for creating reconfigurable light-induced electrodes for fluid manipulation. Since EWOD microfluidics involves direct contact between the droplet and the hydrophobic surface, issues to be considered are droplet (reagent and sample) storage, cross-contamination and loss of sample due to the non-specific adsorption of proteins and other reagents on the dielectric surface. To minimize protein absorption, a low concentration of pluronic additives can be applied in the solution to facilitate fluid actuation with high concentration of proteins [141]. Alternatively, a replaceable, polymeric "skin" strategy has been reported for eliminating cross-contamination and facilitating the "world-to-chip" interface by reagent preloading in the skin [142].

## Electrokinetics

### Background

Electrokinetics is a promising microfluidic technology for biological engineering due to its effectiveness on small scales, label-free manipulation, and well-established techniques for fabricating microelectrodes [143]. DC electrokinetic techniques, such as electrophoresis and electroosmosis, have been intensively studied for protein and nucleic acid analyses since the early stage of microfluidic development. AC electrokinetics including dielectrophoresis, electrothermal flow, and AC electroosmosis has also gained significant interests in the past decade and is emerging as a powerful microfluidic strategy [144,145]. Similar to the EWOD platform, only electronic interfaces are required for electrokinetic manipulation. This is beneficial for developing point-of-care diagnostic systems taking advantage of the recent advancement of portable electronics. Furthermore, multiple electrokinetic phenomena can be combined to perform various microfluidic operations such as mixing, concentration and separation on a single device with low applied AC potential (< 10 V_pp_) [146]. These characteristics make electrokinetics a potential technology for developing fully integrated lab-on-a-chip systems.

### Technology

**Figure 6:**
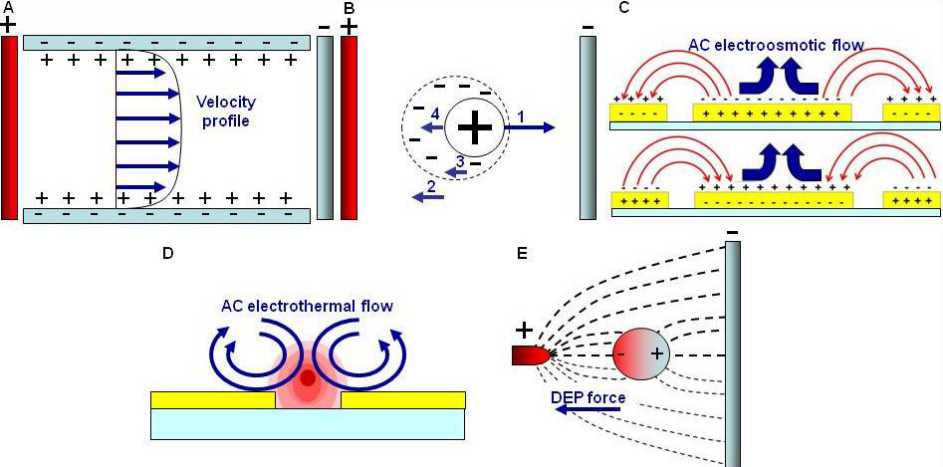
**Electrokinetic platform**. (A) Schematic of the formation of electrical double layer and electroosmotic flow. (B) The four forces acting on a charged particle in the electrophoresis experiment. Force 1 is the electrostatic force between the charged particle and the electric field. Force 2 is the viscous drag. Force 3 is the electrophoretic retardation. Force 4 is the electrophoretic relaxation. (C) Schematic of AC electroosmosis. (D) Schematic of AC electrothermal flow. (E) Schematic of dielectrophoresis.

Electrokinetics is the motion of fluids or embedded objects induced by external electric fields. With DC electric potentials, the two major electrokinetic phenomena observed are electroosmosis [147] and electrophoresis [148]. Electroosmosis is the motion of liquid as a result of the interaction between the applied electric field and the electric double layer. In a microfluidic channel, the charges on the surface attract counter-ions from the solution and repel co-ions resulting in an electric double layer near the surface (Figure 6a). When an external electric field is applied, the charges in the electric double layer experience a net Coulomb force and migrate along the microchannel. The bulk fluid in the channel is then dragged along with the fluid. As a result, the fluid migrates with a uniform velocity profile in the microchannel. It can serve as a pumping mechanism free of mechanical moving parts [149]. By applying a gate voltage to control the zeta potential, dynamic control of electroosmosis including flow reversal and mixing can be achieved and a flow rate of over 1 μL/min has been achieved with a low gate voltage [150]. Another DC electrokinetic phenomenon, electrophoresis, is a particle force acting directly on a charged object. Figure 6b shows the forces acting on a colloidal charged particle suspended in an aqueous electrolyte solution under the influence of the DC electric field. Force 1 is the electrostatic force between the electric surface charge of the particle and the applied electric field [151]. Force 2 is the viscous drag. The electric field also exerts an electrophoretic retardation (force 3) on the counterions in the double layer in a direction opposite to that of the charged particle. The ion movement gives rise to the fluid motion around the particle (electroosmosis), enhancing the viscous drag on the particle. The last one is the electrophoretic relaxation (force 4). Under the influence of the electric field, the force causes the separation between the charged particle and the mobile ions in the double layer. The induced dipole will create an extra drag force on the particle. It should be noted that both the electrophoretic retardation and relaxation forces are a function of the double layer thickness. Therefore, the electrophoretic velocity depends not only on the charge and the size of the particle, but also the properties of the environment. Since most biological molecules, such as DNA and proteins, are charged, electrophoresis can broadly be applied for a variety of biological separation and manipulation processes.

Under AC electric fields, two types of electrohydrodynamic flow, AC electroosmosis and AC electrothermal flow, can be observed on the microscale [152]. In particular, AC electroosmotic flow is the result of interaction between the applied electric field and the electric double layer induced by electrode polarization (Figure 6c). Since the amount of charges induced by the electric field at the interface of the electrode and electrolyte is dependent on the applied frequency, AC electroosmotic flow is also frequency sensitive. At the low frequencies (<100 Hz), since the electric field in the bulk electrolyte is negligible small, AC electroosmotic effect becomes insignificant. At high frequencies (>100 kHz), AC electroosmotic effect is also insignificant because the time is not sufficient for the formation of the electrical double layer. Therefore, AC electroosmosis is effective at intermediate frequencies (e.g., 100 Hz-100 kHz) and for media with relatively low conductivity (< 100 mS/m) [116]. On the other hand, AC electrothermal flow is the result of Joule heating and is effective at high frequencies (e.g., > 100 kHz) [115]. In AC electrothermal flow, the electric field causes power dissipation in the fluid and gives rise to a temperature gradient (Figure 6d). This results in conductivity and permittivity gradients. The electric field acts on these gradients to create a net body force on the fluid. The fluid velocity is proportional to the temperature rise in the fluid, which is in turn proportional to the fluid conductivity. Therefore, electrothermal flow is especially effective in high conductivity buffers. Most of the clinical and physiological fluids have relatively high conductivities [153], which highlights the importance of electrothermal flow in biomedical applications. Another AC electrokinetic effect is dielectrophoresis, which is an electrokinetic force exerted on dielectric particles [115] (Figure 6e). When a dielectric particle is under the influence of an electric field, a dipole moment is induced inside the particle. The dipole then experiences a net force under an electric field gradient with either a spatially varying magnitude or phase. The magnitude and direction of the force depends strongly on the shape and size of the particle, frequency of the electric field and the electrical properties of both the fluid and the particle.

### Applications

Capillary electrophoresis is one of the first DC electrokinetics driven microfluidic platforms. The system typically consists of an electroosmotic pumping system for sample loading and an electrophoretic separation channel for sample analysis [154,155]. This technique can separate charged species based on their size-to-charge ratio. Since the velocity profile in electroosmotic flow is uniform, electrokinetic driven systems can minimize the band broadening as in pressure driven systems. Since the joule heating effect is undesirable for capillary electrophoresis, the separation efficiency of DNA samples has been enhanced when electrophoresis and electroosmotic effects are induced by pulsed DC electric fields, compared to those induced by continuous DC electric fields of the same intensity [156]. Microfluidic platforms for sizing, quantification and quality control of DNA, RNA, proteins and cells on a single platform equipped with flow cytometry and electrophoresis analysis has been demonstrated and commercialized [157]. Other electrokinetic platforms combined with the pressure driven flow system and LED detector for various biochemical applications are also available [158,159]. For instance, a CMOS microarray exploits DC electrophoresis for manipulating molecules and cells has also been demonstrated [160,161]. In this system, the chip contains as many as 400 individually addressable test sites and the electrode is covered with a thin hydrogel permeation layer to prevent water electrolysis and enhanced surface binding. Furthermore, cell analyses including lysis, separation, and detection operated on a DC electrokinetics driven microfluidic platform has been reported [162,163].

**Figure 7:**
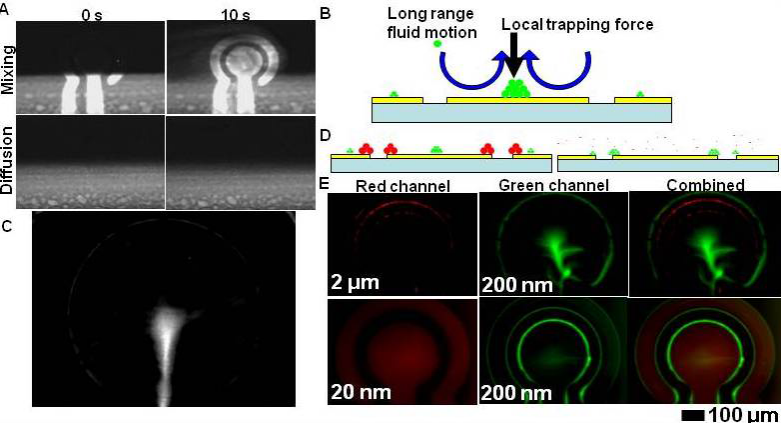
**Hybrid electrokinetics for mixing, separation, and concentration**. (A) Fluorescence images of electrokinetic-induced mixing (top) and diffusion-based mixing (bottom) at the beginning (left) and after 10 sec (right) [146]. (B) Schematic for particle concentration with hybrid electrokinetics. Long range fluid motion drives particles to regions near the electrode where other local electrokinetic forces trap the particles [146]. (C) Concentration nanoparticles on top of the electrode with hybrid electrokinetics [146]. (D) Schematic on the left demonstrates the separation of 200 nm particles (green) from 2 μm particles (red) [146]. Dielectrophoretic force dominates for large particles and traps them at the electrode edge while 200 nm particles are pushed toward the centre of the electrode. Schematic on the right illustrates the separation of 200 nm particles (green) from 20 nm particles (red). At an appropriate frequency, dielectrophoretic force is adequate for trapping 200 nm particles but not for trapping 20 nm particles. (E) Top row shows the separation of 200 nm particles (green) from 2 μm particles (red) [146]. Bottom row shows the separation of 200 nm particles (green) from 20 nm particles (red).

Although existing commercially available products are mainly based on DC electrokinetics, AC electrokinetics has been under rapid development recently. Fundamental microfluidic operations such as mixing [146,164,165] (Figure 7a), pumping [166-168], concentration [169,170] (Figure 7b and 7c) and separation [171-174] (Figure 7d and 7e) based on AC electrokinetics can be performed with low AC voltage, which presents an advantage of AC electrokinetics over DC electrokinetics. Another interesting characteristic of electrokinetics is the fact that the magnitude and direction of various electrokinetic forces depend on a number of inter-related parameters including frequency, magnitude and phase of the electric field, and the physical properties of the fluid and particles. This allows the implementation of multiple electrokinetic phenomena simultaneously on the same electrode platform and the manipulation of particles and fluid with great controllability for performing various fundamental microfluidic operations. Various biochemical analyses and microfluidic operations have been performed with the implementation of multiple AC electrokinetic effects simultaneously. For example, a hybrid electrokinetic bioprocessor has been developed. In this device, the long-range AC electroosmosis can transport embedded particles in the solution to the regions near the electrode surface and the short-range electrophoretic and dielectrophoretic forces effectively trap the target particles on the electrode surface [146,175]. Furthermore, drug delivery [176], cell separation [177] and temporal and signal enhancement for immunoassays have been facilitated with AC electrokinetics.

### Strength and weakness of the platform

In terms of system integration, electrokinetic platforms require only simple microfabrication techniques and various electrokinetic sample preparation modules can be integrated easily on the same chip. While DC electroosmotic flow is attractive for chromatographic separation and analysis, DC electrokinetic platform requires high voltage operation, which could be challenging to implement in a hand-held device or in resource limited settings. On the other hand, AC electrokinetics can be carried out at lower voltage, which makes it a more feasible technique for point-of-care diagnostics. Furthermore, multiple electrokinetic phenomena can be combined to perform various fluidic operations on a single device by adjusting the operating parameters, e.g., frequency and the amplitude of the applied voltage. This renders its broad applicability for a variety of medical diagnostic applications. However, electrokinetics can be limited by electrochemical effects including electrolysis, electrode erosion and sample deterioration. Although electrode erosion can be lessened by coating the electrode array, the electrothermal effect could not be ignored. Strong electrothermal effect may result in damages of the protective layer, sample degradation and evaporation [178]. The electrochemical effect should be considered when designing electrokinetic driven microfluidic systems. Finally, as electrokinetic effects are sensitive to the conductivity of sample fluid, the properties of the sample can affect the operational performance of the platform. In situ characterization of the sample conductivity using impedance spectroscopy or other techniques should be integrated into the microfluidic system for handling samples with unknown conductivities.

## Centrifugal Microfluidics

### Background

The centrifugal microfluidic platform, or Lab-on-a-CD, is another promising system integration technology. Lab-on-a-CD system has been a focus of intense research for years due to the simplicity of the instrumentation interface and capabilities of implementing a wide range of fluidic processing steps, such as pumping, mixing, valving, metering, and routing without the requirement of bulky instruments. These functions allow complete automation for various biochemical analyses. A review on centrifugal microfluidics for biomedical applications has been published recently [179].

### Technology

**Figure 8:**
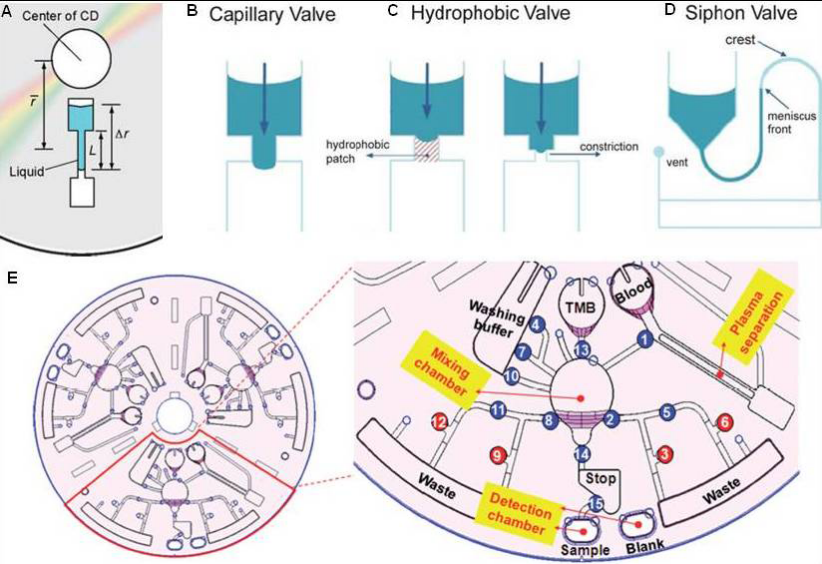
**Centrifugal microfluidics platform**. (A) Centrifugal microfluidics valve is built based on two reservoirs connected by a microfluidic chamber [181]. (B) The capillary valve with a hydrophilic microchannel [179]. (C) The hydrophobic valve [179]. (D) The hydrophilic siphon valve [179]. (E) The CD design illustrating the detailed microfluidic layout and functions for fully automated ELISA system [194]. The number indicates the order of operations.

The processes in centrifugal microfluidics are based on a rotating microstructured substrate controlled by a motor (Figure 8a). In centrifugal microfluidics, a combination of centrifugal force, Coriolis force and capillary force are applied to manipulate the sample. For instance, liquid transport or pumping can be achieved the outward centrifugal forces that direct the sample liquids from the center toward the rim of the disc. The fluid motion can be controlled by the angular velocity, flow resistance of the channels and the properties of the sample, e.g., viscosity and density. By controlling the rotational frequency and channel geometries, a dynamic range of flow rates from nL/s to mL/s can be generated [180,181]. Unlike electrokinetics or EWOD platforms, the flow motions are relatively insensitive to the conductivities, pH, and chemical compositions of the liquids. This allows the operation of a wide range of fluids on this platform.

Valving in centrifugal microfluidics can be constructed as passive valve or active value on the disc. The most widely used design is the capillary valve, which is a sudden expansion of the microfluidic channel [182] (Figure 8b). Liquid flow stops when the centrifugal pressure is equal to or less than the capillary barrier pressure. The valve will open when the rotational frequency exceeds the critical burst frequency, which depends on the surface tension and geometric parameters of the channel. The second type of valve is the hydrophobic valve. The fluid flow can be impeded when part of the channel is functionalized with hydrophobic material or when there is a sudden constriction in the hydrophobic channel (Figure 8c). The third method is based on the siphon structure (Figure 8d). When the rotational speed is high, two liquid-gas interfaces are at the same level because of the centrifugal force (valve closes) [183]. Below the critical frequency, the meniscus front on the right passes beyond the bend and fills the channel (valve opens). The siphon valve provides valving at higher spin speeds while the capillary operates at lower spin speeds. There are two drawbacks related to the passive valves discussed above. Firstly, they are not vapor-tight, which could potentially cause cross-contamination among reagents if they have high vapor pressure or high temperature process is involved for the reagents. Secondly, the rotational motion must be adjusted from slow to fast, not vice versa. Alternatively, an active valve has recently been reported which is based on an irreversible and one-time use process [184]. The valve is composed of iron oxide nanoparticles dispersed in paraffin wax. When a laser bean excites the valves, the nanoparticles are able to couple with the laser energy for melting the wax. The valve can be designed in both normally open and close states. In the normally open valve, the chamber preloaded with ferrowax is built adjacent to the main channel. Molten wax, irradiated by laser, bursts into the main channel and solidifies, which in turn blocks the channel. In a normally closed valve, the ferrowax plug is located between two chambers and blocks the fluid flow. After applying the laser power, molten wax flows to the chamber and solidifies, which results in the opening of the channel. This active valve is independent of the spin speed. Aliquoting of liquids has also been reported based on the valving principle [185]. The device for aliquoting consists of an upstream metering channel and a downstream unvented reaction chamber separated by a narrow connection channel. Fluid entering the metering channel seals the reaction chamber. When the rotational speed is increased above the critical burst frequency, the fluid fills the reaction chamber. The metering volumes are set by the capacity of the reaction chamber.

One of the techniques for mixing in centrifugal microfluidics, the shake-mode mixing, involves rapid oscillation of the disc between clockwise and counter clockwise directions [186]. The inertia of the liquid induces a gradient of angular momentum to promote mixing. This strategy can reduce the mixing times from 7 minutes with mere diffusion to several seconds with the shake-mode mixing. Other mixer designs have also been demonstrated based on this principle. For instance, unidirectional shake-mode mixing has been developed for minimizing the valving problems with buffers of high detergent or salt concentrations [187]. In this approach, the disc was accelerated and decelerated sequentially while the spinning direction stayed the same [188]. This technique is capable of mixing 30 μl of fluid in less than 3 min.

### Applications

A wide range of applications have been realized using the centrifugal microfluidic platform. For example, the versatile platform allows extraction of plasma from whole blood [189] and nucleic acid from clinical samples, such as nasopharyngeal aspirates [190]. Dielectrophoresis assisted selective filtering with 3D carbon electrodes fabricated in a centrifugal platform has also been reported [191]. Using this system, a mixture of latex particles and yeast cells can be isolated by trapping the yeast cells at flow rates up to 35 μl/min. Fully automated nucleic acid analysis systems based on real-time polymerase chain reaction (PCR) [192] or isothermal recombinase polymerase amplification (RPA) [193] is another successful application of centrifugal microfluidics. A limit of detection below 10 copies of DNA has been achieved using the system. Other applications include infectious agent detection from blood with enzyme-linked immuno-sorbent assay (ELISA) [194] (Figure 8e), on-site pre-concentration and screening of organic contaminants in aqueous samples [195], and extraction of pathogen specific DNA from the whole blood.

Various biological assays can be incorporated in the centrifugal microfluidic platform [196]. Examples of assay formats include sandwich immunoassay for antigen quantification, indirect antibody immunoassay and bridging immunoassay for antibody quantification. To perform the assay, streptavidin-coated microparticles can be first pre-packed into the system. The binding of the target molecules (antigens or antibodies) proceeds after the mixing the microparticles with the biotin-labeled capture reagent. A laser induced fluorescent (LIF) detector integrated with the workstation can detect the analyte with the fluorophore-labeled complex. Multiple immunoassays have been carried out at the nanoliter scale and 112 column scans can be accomplished in only 1.5 mins. Immunoassays including biomarkers detection such as cytokines, pharmacokinetics (PK)/toxicokinetics (TK) assays and anti-drug antibody (ADA) assays have been demonstrated with the technique. Another CD based platform, Piccolo Xpress, is capable of processing whole blood samples with only 0.1 cc sample size and the results are available in 12 minutes [197].

### Strength and weakness of the platform

There are several advantages of the centrifugal microfluidic platform. Firstly, it requires only a simple and compact motor to create rotational motion for fluid manipulation. The microfluidic elements imprinted on the single disc can perform all basic microfluidic operations required for a fully automated system. Multiplexed analyses can be achieved due to the rotational symmetry of the disks. Additionally, the fabrication of the centrifugal microfluidic system is cost-effective since large scales of plastics cartridges can be made at low-cost. However, due to the rotational motion, contact free interface is required for other additional modules such as optical detection and actuation. Another consideration is that as the whole disc is rotated at the same frequency, processes with different critical frequencies are difficult to be implemented simultaneously. Lastly, once all the elements are imprinted on the cartridge permanently, the platform offers little reconfigurability seeing that the re-design of new channels are needed for each assay.

## Future Directions

**Figure 9:**
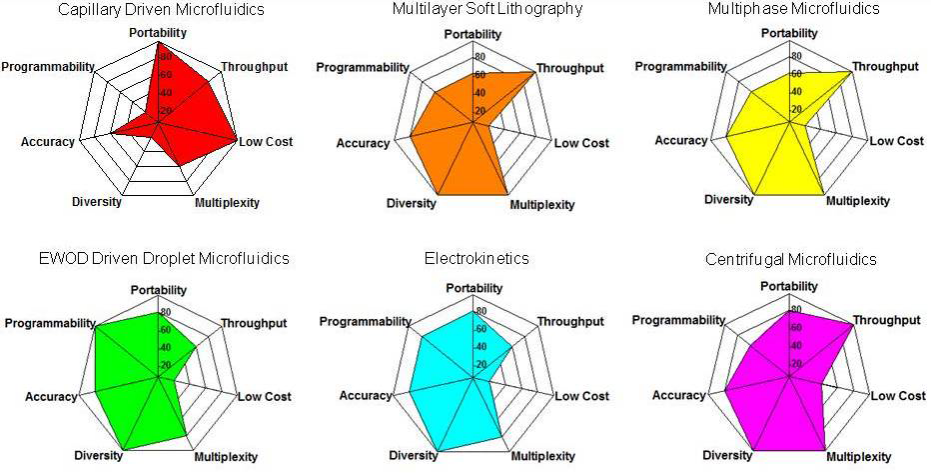
**Characteristics of different microfluidics platforms**. Comparisons of key selection criteria for different microfluidic system integration strategies. The properties include portability, the number of samples analyzed in a single assay (throughput), the cost of the instrument, the number of parameters tested for each sample (multiplexity), variety of microfluidic operations (diversity), accuracy, and the flexibility to comply different microfluidic operations without making a new chip (programmability). The higher the rating implies the better performance of the platform in the specific property.

Recently, microfluidic researchers have devoted a large amount of effort to develop microfluidic platforms from a system-oriented rather than components-oriented perspective. Not only can they provide a set of basic microfluidic operating procedures but also allow easy interface of these fluidic operation modules. Each platform possesses its own strengths and weaknesses in terms of several general quality criteria for the realization of lab-on-a-chip (Figure 9). These important characteristics include portability, the number of samples that can be analyzed in a single assay (throughput), the cost of the instrument, the number of parameters tested for each sample (multiplexity), variety of microfluidic operations (diversity), accuracy, and the flexibility to implement complex microfluidic operations for different applications (programmability). The importance of these criteria depends on the specific application being considered and the list of criteria can serve as a general guideline for the selection of a microfluidic system integration strategy.

For applications with complicated tasks, such as concentration, mixing and separation, hybrid microfluidics, which is the incorporation of different microfluidic strategies into a single platform, can be a solution for maintaining the portability and simplicity of the system. For example, electrokinetic technologies such as dielectrophoresis and electrophoresis have been integrated into the EWOD platform for particle concentration and separation within the droplets [129,130]. Another example involves the fabrication of 3D carbon electrodes in a centrifugal platform for trapping particles of interest [191]. Electrokinetic approaches have also been widely adopted in the multiphase microfluidics platform for droplet fusion and sorting [110-112]. Another hybrid microfluidics configuration, which is the integration of EWOD with microchannels on a single platform, has also been demonstrated for in-line sample processing and separations [198]. The hybrid microfluidics approach will be a prospective strategy for obtaining a more flexible and cost-efficient chip design as it can take advantage of the full potential of the microfluidics platform and at the same time alleviate its limitations with other microfluidics technologies.

Beside the fluidic components for performing basic fluidic operations, the detection module for converting biochemical responses into quantifiable signals is another crucial element in a lab-on-a-chip system. Most of the biomedical applications require sensitive detection modules, such as microscopes for cell, bacteria, and fluorescence visualization, thermal cyclers for PCR reactions, and mass spectrometers for sample analyses. This is one of the major reasons of why many commercially available lab-on-a-chip systems are limited to bench-top rather than handheld instruments. Therefore, there is a growing interest for the microfluidics community to investigate portable detection modules. By now, the two most prevalent types of sensing modules with the microfluidic systems are based on optical and electrical signals. For optical detection, the most commercially successful module for system integration is certainly the lateral flow tests in which the readout of an assay is mostly implemented with manual observation of the color change in the detection zone. However, manual observation is only applicable to applications with high analyte concentrations. For most biochemical applications, advanced detection strategies, such as bright field and dark field imaging, confocal microscopy, laser induced fluorescence microscopy and surface plasmon resonance microscopy, are required due to their high sensitivity, resolution, and signal-to-noise ratio [199-203]. To avoid bulky optics, the development of simple, miniaturized, and low-cost optical systems is a major research area recently [204-209]. For example, lensfree digital microscopy has been demonstrated with compact, light-weight and cost effective optical components mechanically attached to a camera unit of a cell phone [210]. Images of micro-sized objects such as red blood cells, white blood cells, and platelets can be captured with the system. This approach will also be useful in promoting global health delivery through telecommunication. For electrical detection modules, electrochemistry is a promising candidate for lab-on-a-chip devices. Not only because of its high sensitivity, but also the electrical signal can be processed by conventional electronics and the miniaturization and integration of the electrochemical transducer into a microfluidic device is feasible [211]. As of now, the adaptation of electrochemistry as the sensing modules has been realized for detecting various types of pathogens and biomolecules, such as glucose, lactate, uric acid [212], anti-DNA antibodies [213], and uropathogens.

Over the past ten years, although academic and industrial researchers have put a lot of effort on developing different microfluidic devices, it still has not gained widespread market adoption by the general public or even by the research community [214]. Interestingly, the development of a successful technology is often driven by only one or two important applications (i.e., an killer app), especially for those technologies requiring large capital investments [214]. For instance, MEMS accelerometer is primarily driven by the automobile industry while it is currently adopted in various consumer electronics, such as digital camera and video game systems, and defense applications. From this view point, the field of microfluidics is in search for an important application to drive the commercialization of the field. It will likely be an application that has a large demand and can justify the cost of a microfluidic diagnostic device. These characteristics are required to justify the risk and large scale investment associated with commercializing a microfluidic system. Potential candidates include detection systems for infectious diseases (e.g., urinary tract infection, human immunodeficiency virus, diarrheal diseases, and tuberculosis), cardiac markers for risky heart attack patients, early stage cancer diagnostics, and bio/chemical warfare agents for security applications.

## Conclusions

Numerous ongoing research works are working on building novel microfluidic platforms through the development of novel system integration strategies. Further development in this area will lead to fully automated microfactories that allow various biochemical analyses to be performed at low cost for a wide spectrum of biological and biomedical engineering applications [114,215-218].

## Competing interests

The authors declare that they have no competing interests.

## Authors' contributions

MLYS and PKW reviewed fundamental microfluidics platforms and drafted the manuscript. GJ and JCL reviewed commercial and clinical applications of microfluidic platforms. All authors contributed to the future directions. All authors read and approved the final manuscript.

## References

[B1] Whitesides GM The origins and the future of microfluidics Nature 2006 442 368 373 10.1038/nature05058 16871203

[B2] Chen CH Lu Y Sin MLY Mach KE Zhang DD Gau V Liao JC Wong PK Antimicrobial Susceptibility Testing Using High Surface-to-Volume Ratio Microchannels Analytical Chemistry 2010 82 1012 1019 10.1021/ac9022764 20055494 PMC2821038

[B3] Squires TM Quake SR Microfluidics: Fluid physics at the nanoliter scale Rev Mod Phys 2005 77 977 1026 10.1103/RevModPhys.77.977

[B4] Lam MHC Homenuke MA Michal CA Hansen CL Sub-nanoliter nuclear magnetic resonance coils fabricated with multilayer soft lithography J Micromech Microeng 2009 19 095001 10.1088/0960-1317/19/9/095001

[B5] Chung AJ Kim D Erickson D Electrokinetic microfluidic devices for rapid, low power drug delivery in autonomous microsystems Lab on a Chip 2008 8 330 338 10.1039/b713325a 18231674

[B6] Sista R Hua ZS Thwar P Sudarsan A Srinivasan V Eckhardt A Pollack M Pamula V Development of a digital microfluidic platform for point of care testing Lab on a Chip 2008 8 2091 2104 10.1039/b814922d 19023472 PMC2726010

[B7] Hettiarachchi K Talu E Longo ML Dayton PA Lee AP On-chip generation of microbubbles as a practical technology for manufacturing contrast agents for ultrasonic imaging Lab on a Chip 2007 7 463 468 10.1039/b701481n 17389962 PMC2253663

[B8] Rohde CB Zeng F Gonzalez-Rubio R Angel M Yanik MF Microfluidic system for on-chip high-throughput whole-animal sorting and screening at subcellular resolution Proceedings of the National Academy of Sciences of the United States of America 2007 104 13891 13895 10.1073/pnas.0706513104 17715055 PMC1955819

[B9] Riegger L Grumann M Steigert J Lutz S Steinert CP Mueller C Viertel J Prucker O Ruhe J Zengerle R Ducree J Single-step centrifugal hematocrit determination on a 10-$ processing device Biomedical Microdevices 2007 9 795 799 10.1007/s10544-007-9091-1 17534715

[B10] Chikkaveeraiah BV Liu HY Mani V Papadimitrakopoulos F Rusling JF A microfluidic electrochemical device for high sensitivity biosensing: Detection of nanomolar hydrogen peroxide Electrochemistry Communications 2009 11 819 822 10.1016/j.elecom.2009.02.002 20161158 PMC2735753

[B11] Lee W Fon W Axelrod BW Roukes ML High-sensitivity microfluidic calorimeters for biological and chemical applications Proceedings of the National Academy of Sciences of the United States of America 2009 106 15225 15230 10.1073/pnas.0901447106 19706406 PMC2741232

[B12] Blanco-Gomez G Glidle A Flendrig LM Cooper JM Integration of Low-Power Microfluidic Pumps with Biosensors within a Laboratory-on-a-Chip Device Analytical Chemistry 2009 81 1365 1370 10.1021/ac802006d 19143543

[B13] Chang ST Beaumont E Petsev DN Velev OD Remotely powered distributed microfluidic pumps and mixers based on miniature diodes Lab on a Chip 2008 8 117 124 10.1039/b712108c 18094769

[B14] Mair DA Schwei TR Dinio TS Svec F Frechet JMJ Use of photopatterned porous polymer monoliths as passive micromixers to enhance mixing efficiency for on-chip labeling reactions Lab on a Chip 2009 9 877 883 10.1039/b816521a 19294297 PMC2790067

[B15] Rosenfeld C Serra C Brochon C Hadziioannou G Influence of micromixer characteristics on polydispersity index of block copolymers synthesized in continuous flow microreactors Lab on a Chip 2008 8 1682 1687 10.1039/b803885f 18813391

[B16] Zhang CS Xing D Li YY Micropumps, microvalves, and micromixers within PCR microfluidic chips: Advances and trends Biotechnol Adv 2007 25 483 514 10.1016/j.biotechadv.2007.05.003 17601695

[B17] Gao J Sin MLY Liu T Gau V Liao JC Wong PK Hybrid Electrokinetic Manipulation in High-Conductivity Media Lab Chip 2011 11 1770 1775 10.1039/c1lc20054b 21487576 PMC4084846

[B18] Churski K Michalski J Garstecki P Droplet on demand system utilizing a computer controlled microvalve integrated into a stiff polymeric microfluidic device Lab on a Chip 2010 10 512 518 10.1039/b915155a 20126693

[B19] Kaigala GV Hoang VN Backhouse CJ Electrically controlled microvalves to integrate microchip polymerase chain reaction and capillary electrophoresis Lab on a Chip 2008 8 1071 1078 10.1039/b802853b 18584081

[B20] Kim JT Chen DF Bau HH An automated, pre-programmed, multiplexed, hydraulic microvalve Lab on a Chip 2009 9 3594 3598 10.1039/b914865e 20024041 PMC12093303

[B21] Mark D Haeberle S Roth G von Stetten F Zengerle R Microfluidic lab-on-a-chip platforms: requirements, characteristics and applications Chemical Society Reviews 2010 39 1153 1182 10.1039/b820557b 20179830

[B22] Mariella R Sample preparation: the weak link in microfluidics-based biodetection Biomedical Microdevices 2008 10 777 784 10.1007/s10544-008-9190-7 18483862 PMC7088034

[B23] Kim DH Wong PK Park J Levchenko A Sun Y Microengineered Platforms for Cell Mechanobiology Annual Review of Biomedical Engineering 2009 11 203 233 10.1146/annurev-bioeng-061008-124915 19400708

[B24] Kim J Junkin M Kim DH Kwon S Shin YS Wong PK Gale BK Applications, techniques, and microfluidic interfacing for nanoscale biosensing Microfluidics and Nanofluidics 2009 7 149 167 10.1007/s10404-009-0431-8

[B25] Wang TH Peng YH Zhang CY Wong PK Ho CM Single-molecule tracing on a fluidic microchip for quantitative detection of low-abundance nucleic acids Journal of the American Chemical Society 2005 127 5354 5359 10.1021/ja042642i 15826173

[B26] Lawi W Wiita C Snyder ST Wei F Wong D Wong PK Liao JC Haake D Gau V A Microfluidic Cartridge System for Multiplexed Clinical Analysis Jala-J Assoc Lab Aut 2009 14 407 412 10.1016/j.jala.2009.05.002 PMC2808045 20161584

[B27] Chiu ML Lawi W Snyder ST Wong PK Liao JC Gau V Matrix Effect - A Challenge Toward Automation of Molecular Analysis Journal of Association for Laboratory Automation 2010 15 233 242 10.1016/j.jala.2010.02.001

[B28] Choi S Chae J A Physisorbed Interface Design of Biomolecules for Selective and Sensitive Protein Detection Journal of Association for Laboratory Automation 2010 15 172 178 10.1016/j.jala.2009.09.002

[B29] Garcia DE Chen T-H Wei F Ho CM A Parametric Design Study of an Electrochemical Sensor Journal of Association for Laboratory Automation 2010 15 179 188 10.1016/j.jala.2010.01.007

[B30] Kwong H-J Dean ZS Angus SV Yoon J-Y Lab-on-a-Chip for Field Escherichia coli Assays: Long-Term Stability of Reagents and Automatic Sampling System Journal of Association for Laboratory Automation 2010 15 216 226 10.1016/j.jala.2010.01.011

[B31] Lawi W Wiita C Snyder ST Wei F Wong D Wong PK Liao JC Haake DA Gau V A Microfluidic Cartridge System for Multiplexed Clinical Analysis Journal of Association for Laboratory Automation 2009 14 407 412 10.1016/j.jala.2009.05.002 PMC2808045 20161584

[B32] Li N Detection of Non-Nucleic Acid Targets With an Unmodified Aptamer and a Fluorogenic Competitor Journal of Association for Laboratory Automation 2010 15 189 197 10.1016/j.jala.2010.02.002 PMC2885824 20563298

[B33] Lin H-C Liu Y-J Yao D-J Core-Shell Droplets for Parallel DNA Ligation of an Ultra-micro Volume Using an EWOD Microfluidic System Journal of Association for Laboratory Automation 2010 15 210 215 10.1016/j.jala.2010.01.010

[B34] Mandy LY Sin VG Liao Joseph C Wong Pak Kin Electrothermal Fluid Manipulation of High-Conductivity Samples for Laboratory Automation Applications Journal of Association for Laboratory Automation 2010 15 426 432 10.1016/j.jala.2010.05.004 PMC3003926 21180401

[B35] Ryu K Chung SK Cho SK Micropumping by an Acoustically Excited Oscillating Bubble for Automated Implantable Microfluidic Devices Journal of Association for Laboratory Automation 2010 2010 163 171

[B36] Sin MLY Gau V Liao JC Wong PK Electrothermal Fluid Manipulation of High-Conductivity Samples for Laboratory Automation Applications Journal of Association for Laboratory Automation 2010 15 426 432 10.1016/j.jala.2010.05.004 PMC3003926 21180401

[B37] Wang TH Wong PK Transforming Microfluidics into Laboratory Automation Journal of Association for Laboratory Automation 2010 15 A15 A16 10.1016/j.jala.2010.03.002

[B38] Wang Z Gidwani V Sun Z Zhang DD Wong PK Development of a molecular assay for rapid screening of chemopreventive compounds targeting Nrf2 Journal of Association for Laboratory Automation 2008 13 243 248 10.1016/j.jala.2008.03.007

[B39] Yang W T WA Integrated Multiprocess Microfluidic Systems for Automating Analysis Journal of Association for Laboratory Automation 2010 15 198 209 10.1016/j.jala.2010.01.008 PMC2877209 20514343

[B40] Chin CD Linder V Sia SK Lab-on-a-chip devices for global health: Past studies and future opportunities Lab on a Chip 2007 7 41 57 10.1039/b611455e 17180204

[B41] Yager P Edwards T Fu E Helton K Nelson K Tam MR Weigl BH Microfluidic diagnostic technologies for global public health Nature 2006 442 412 418 10.1038/nature05064 16871209

[B42] Yager P Domingo GJ Gerdes J Point-of-care diagnostics for global health Annual Review of Biomedical Engineering 2008 10 107 144 10.1146/annurev.bioeng.10.061807.160524 18358075

[B43] Mach KE Wong PK Liao JC Biosensor Diagnosis of Urinary Tract Infection: A Path to Better Treatment? Trends in Pharmacological Sciences (accepted) 10.1016/j.tips.2011.03.001 PMC3106133 21458868

[B44] Lee WG Kim YG Chung BG Demirci U Khademhosseini A Nano/Microfluidics for diagnosis of infectious diseases in developing countries Adv Drug Deliver Rev 2010 62 449 457 10.1016/j.addr.2009.11.016 PMC2829381 19954755

[B45] Kim J Junkin M Kim DH Kwon S Shin YS Wong PK Gale BK Applications, Techniques, and Microfluidic Interfacing for Nanoscale Biosensing Microfluidics and Nanofluidics 2009 7 149 167 10.1007/s10404-009-0431-8

[B46] Whitesides G Solving problems Lab on a Chip 2010 10 2317 2318 20717619 10.1039/c0lc90036b

[B47] Haeberle S Zengerle R Microfluidic platforms for lab-on-a-chip applications Lab on a Chip 2007 7 1094 1110 10.1039/b706364b 17713606

[B48] Lateral Flow Immunoassay 2009 United States: Springer

[B49] Schwartmann B Enbergs H Results and Practical Experiences with an Early-Pregnancy Diagnosis for Mares Using a Rapid Radioimmunoassay for Progesterone Detection in Blood Zuchthygiene-Reproduction in Domestic Animals 1980 15 75 75

[B50] Martinez AW Phillips ST Wiley BJ Gupta M Whitesides GM FLASH: A rapid method for prototyping paper-based microfluidic devices Lab on a Chip 2008 8 2146 2150 10.1039/b811135a 19023478 PMC3065062

[B51] Posthuma-Trumpie GA Korf J van Amerongen A Lateral flow (immuno) assay: its strengths, weaknesses, opportunities and threats. A literature survey Anal Bioanal Chem 2009 393 569 582 10.1007/s00216-008-2287-2 18696055

[B52] Shimojo N Naka K Nakajima C Yoshikawa C Okuda K Okada K Test-Strip Method for Measuring Lactate in Whole-Blood Clinical Chemistry 1989 35 1992 1994 2776334

[B53] Zimmermann M Hunziker P Delamarche E Autonomous capillary system for one-step immunoassays Biomedical Microdevices 2009 11 1 8 10.1007/s10544-008-9187-2 18810643

[B54] Gervais L Delamarche E Toward one-step point-of-care immunodiagnostics using capillary-driven microfluidics and PDMS substrates Lab on a Chip 2009 9 3330 3337 10.1039/b906523g 19904397

[B55] Collison ME Stout PJ Glushko TS Pokela KN Mullins-Hirte DJ Racchini JR Walter MA Mecca SP Rundquist J Allen JJ Hilgers ME Hoegh TB Analytical characterization of electrochemical biosensor test strips for measurement of glucose in low-volume interstitial fluid samples Clinical Chemistry 1999 45 1665 1673 10471681

[B56] Martinez AW Phillips ST Whitesides GM Carrilho E Diagnostics for the Developing World: Microfluidic Paper-Based Analytical Devices Analytical Chemistry 2010 82 3 10 10.1021/ac9013989 20000334

[B57] Li X Tian JF Shen W Progress in patterned paper sizing for fabrication of paper-based microfluidic sensors Cellulose 2010 17 649 659 10.1007/s10570-010-9401-2

[B58] Li X Tian JF Nguyen T Shen W Paper-Based Microfluidic Devices by Plasma Treatment Analytical Chemistry 2008 80 9131 9134 10.1021/ac801729t 19551982

[B59] Lu Y Shi WW Qin JH Lin BC Fabrication and Characterization of Paper-Based Microfluidics Prepared in Nitrocellulose Membrane By Wax Printing Analytical Chemistry 2010 82 329 335 10.1021/ac9020193 20000582

[B60] Carrilho E Phillips ST Vella SJ Martinez AW Whitesides GM Paper Microzone Plates Analytical Chemistry 2009 81 5990 5998 10.1021/ac900847g 19572563

[B61] Martinez AW Phillips ST Whitesides GM Three-dimensional microfluidic devices fabricated in layered paper and tape Proceedings of the National Academy of Sciences of the United States of America 2008 105 19606 19611 10.1073/pnas.0810903105 19064929 PMC2604941

[B62] Helton KL Nelson KE Fu E Yager P Conditioning saliva for use in a microfluidic biosensor Lab on a Chip 2008 8 1847 1851 10.1039/b811150b 18941684

[B63] Hatch A Kamholz AE Hawkins KR Munson MS Schilling EA Weigl BH Yager P A rapid diffusion immunoassay in a T-sensor Nature Biotechnology 2001 19 461 465 10.1038/88135 11329017

[B64] Osborn JL Lutz B Fu E Kauffman P Stevens DY Yager P Microfluidics without pumps: reinventing the T-sensor and H-filter in paper networks Lab on a Chip 2010 10 2659 2665 10.1039/c004821f 20680208 PMC4892122

[B65] Levine RL Fromm RE Mojtahedzadeh M Baghaie AA Opekun AR Equivalence of Litmus Paper and Intragastric Ph Probes for Intragastric Ph Monitoring in the Intensive-Care Unit Critical Care Medicine 1994 22 945 948 10.1097/00003246-199406000-00011 7911416

[B66] Huh D Mills KL Zhu X Burns MA Thouless MD Takayama S Tuneable elastomeric nanochannels for nanofluidic manipulation Nature Mater 2007 6 424 428 10.1038/nmat1907 17486084

[B67] Hegg MC Chia-Jean W Lin LY Parviz BA Chia-Jean W Nano-scale quantum dot optical transducers by self-assembly Lasers and Electro-Optics Society, 2005 LEOS 2005 The 18th Annual Meeting of the IEEE 2005 124 125

[B68] Ellerbee AK Phillips ST Siegel AC Mirica KA Martinez AW Striehl P Jain N Prentiss M Whitesides GM Quantifying Colorimetric Assays in Paper-Based Microfluidic Devices by Measuring the Transmission of Light through Paper Analytical Chemistry 2009 81 8447 8452 10.1021/ac901307q 19722495

[B69] Khan MS Thouas G Shen W Whyte G Garnier G Paper Diagnostic for Instantaneous Blood Typing Analytical Chemistry 2010 82 4158 4164 10.1021/ac100341n 20415489

[B70] Klasner SA Price AK Hoeman KW Wilson RS Bell KJ Culbertson CT Paper-based microfluidic devices for analysis of clinically relevant analytes present in urine and saliva Anal Bioanal Chem 2010 397 1821 1829 10.1007/s00216-010-3718-4 20425107

[B71] Martinez AW Phillips ST Carrilho E Thomas SW Sindi H Whitesides GM Simple telemedicine for developing regions: Camera phones and paper-based microfluidic devices for real-time, off-site diagnosis Analytical Chemistry 2008 80 3699 3707 10.1021/ac800112r 18407617 PMC3761971

[B72] Melin J Quake SR Microfluidic large-scale integration: The evolution of design rules for biological automation Annu Rev Bioph Biom 2007 36 213 231 10.1146/annurev.biophys.36.040306.132646 17269901

[B73] Unger MA Chou HP Thorsen T Scherer A Quake SR Monolithic microfabricated valves and pumps by multilayer soft lithography Science 2000 288 113 116 10.1126/science.288.5463.113 10753110

[B74] Regehr KJ Domenech M Koepsel JT Carver KC Ellison-Zelski SJ Murphy WL Schuler LA Alarid ET Beebe DJ Biological implications of polydimethylsiloxane-based microfluidic cell culture Lab on a Chip 2009 9 2132 2139 10.1039/b903043c 19606288 PMC2792742

[B75] Pearce TM Williams JC Microtechnology: Meet neurobiology Lab on a Chip 2007 7 30 40 10.1039/b612856b 17180203

[B76] Oh KW Ahn CH A review of microvalves J Micromech Microeng 2006 16 R13 R39 10.1088/0960-1317/16/5/R01

[B77] Grover WH Ivester RHC Jensen EC Mathies RA Development and multiplexed control of latching pneumatic valves using microfluidic logical structures Lab on a Chip 2006 6 623 631 10.1039/b518362f 16652177

[B78] Yang YN Hsiung SK Lee GB A pneumatic micropump incorporated with a normally closed valve capable of generating a high pumping rate and a high back pressure Microfluidics and Nanofluidics 2009 6 823 833 10.1007/s10404-008-0356-7

[B79] Jeong OC Konishi S Fabrication of a peristaltic micro pump with novel cascaded actuators J Micromech Microeng 2008 18 025022 10.1088/0960-1317/18/2/025022

[B80] Huang SB Wu MH Cui ZF Cui Z Lee GB A membrane-based serpentine-shape pneumatic micropump with pumping performance modulated by fluidic resistance J Micromech Microeng 2008 18 045008 10.1088/0960-1317/18/4/045008

[B81] Bontoux N Dauphinot L Vitalis T Studer V Chen Y Rossier J Potier MC Integrating whole transcriptome assays on a lab-on-a-chip for single cell gene profiling Lab on a Chip 2008 8 443 450 10.1039/b716543a 18305863

[B82] Mosadegh B Kuo CH Tung YC Torisawa YS Bersano-Begey T Tavana H Takayama S Integrated elastomeric components for autonomous regulation of sequential and oscillatory flow switching in microfluidic devices Nat Phys 2010 6 433 437 20526435 10.1038/nphys1637 PMC2880544

[B83] Bhat S Herrmann J Armishaw P Corbisier P Emslie KR Single molecule detection in nanofluidic digital array enables accurate measurement of DNA copy number Anal Bioanal Chem 2009 394 457 467 10.1007/s00216-009-2729-5 19288230

[B84] Kato M Kawaguchi T Ishikawa S Umeda T Nakamichi R Shapero MH Jones KW Nakamura Y Aburatani H Tsunoda T Population-genetic nature of copy number variations in the human genome Human Molecular Genetics 2010 19 761 773 10.1093/hmg/ddp541 19966329 PMC2816609

[B85] Qin J Jones RC Ramakrishnan R Studying copy number variations using a nanofluidic platform Nucleic Acids Research 2008 36 e116 10.1093/nar/gkn518 18710881 PMC2566873

[B86] Spurgeon SL Jones RC Ramakrishnan R High Throughput Gene Expression Measurement with Real Time PCR in a Microfluidic Dynamic Array Plos One 2008 3 e1662 10.1371/journal.pone.0001662 18301740 PMC2244704

[B87] Tay S Hughey JJ Lee TK Lipniacki T Quake SR Covert MW Single-cell NF-kappa B dynamics reveal digital activation and analogue information processing Nature 2010 466 267 U149 10.1038/nature09145 20581820 PMC3105528

[B88] Gomez-Sjoberg R Leyrat AA Pirone DM Chen CS Quake SR Versatile, fully automated, microfluidic cell culture system Analytical Chemistry 2007 79 8557 8563 10.1021/ac071311w 17953452

[B89] Balagadde FK You LC Hansen CL Arnold FH Quake SR Long-term monitoring of bacteria undergoing programmed population control in a microchemostat Science 2005 309 137 140 10.1126/science.1109173 15994559

[B90] Diercks AH Ozinsky A Hansen CL Spotts JM Rodriguez DJ Aderem A A microfluidic device for multiplexed protein detection in nano-liter volumes Analytical Biochemistry 2009 386 30 35 10.1016/j.ab.2008.12.012 19133224 PMC2678059

[B91] Voelkerding KV Dames S Durtschi JD Next Generation Sequencing for Clinical Diagnostics-Principles and Application to Targeted Resequencing for Hypertrophic Cardiomyopathy A Paper from the 2009 William Beaumont Hospital Symposium on Molecular Pathology Journal of Molecular Diagnostics 2010 12 539 551 10.2353/jmoldx.2010.100043 20805560 PMC2928417

[B92] Huang SB Wu MH Lee GB A tunable micro filter modulated by pneumatic pressure for cell separation Sensors and Actuators B-Chemical 2009 142 389 399 10.1016/j.snb.2009.07.046

[B93] Teh SY Lin R Hung LH Lee AP Droplet microfluidics Lab on a Chip 2008 8 198 220 10.1039/b715524g 18231657

[B94] Griffiths AD Tawfik DS Miniaturising the laboratory in emulsion droplets Trends in Biotechnology 2006 24 395 402 10.1016/j.tibtech.2006.06.009 16843558

[B95] Garstecki P Fuerstman MJ Stone HA Whitesides GM Formation of droplets and bubbles in a microfluidic T-junction - scaling and mechanism of break-up Lab on a Chip 2006 6 437 446 10.1039/b510841a 16511628

[B96] Tan YC Cristini V Lee AP Monodispersed microfluidic droplet generation by shear focusing microfluidic device Sensors and Actuators B-Chemical 2006 114 350 356 10.1016/j.snb.2005.06.008

[B97] Stan CA Tang SKY Whitesides GM Independent Control of Drop Size and Velocity in Microfluidic Flow-Focusing Generators Using Variable Temperature and Flow Rate Analytical Chemistry 2009 81 2399 2402 10.1021/ac8026542 19209912

[B98] Nie ZH Seo MS Xu SQ Lewis PC Mok M Kumacheva E Whitesides GM Garstecki P Stone HA Emulsification in a microfluidic flow-focusing device: effect of the viscosities of the liquids Microfluidics and Nanofluidics 2008 5 585 594

[B99] Hung LH Choi KM Tseng WY Tan YC Shea KJ Lee AP Alternating droplet generation and controlled dynamic droplet fusion in microfluidic device for CdS nanoparticle synthesis Lab on a Chip 2006 6 174 178 10.1039/b513908b 16450024

[B100] Seo M Paquet C Nie ZH Xu SQ Kumacheva E Microfluidic consecutive flow-focusing droplet generators Soft Matter 2007 3 986 992 10.1039/b700687j 32900048

[B101] Liu K Ding HJ Chen Y Zhao XZ Droplet-based synthetic method using microflow focusing and droplet fusion Microfluidics and Nanofluidics 2007 3 239 243 10.1007/s10404-006-0121-8

[B102] Adamson DN Mustafi D Zhang JXJ Zheng B Ismagilov RF Production of arrays of chemically distinct nanolitre plugs via repeated splitting in microfluidic devices Lab on a Chip 2006 6 1178 1186 10.1039/b604993a 16929397 PMC1851925

[B103] Menetrier-Deremble L Tabeling P Droplet breakup in microfluidic junctions of arbitrary angles Physical Review E 2006 74 035303 10.1103/PhysRevE.74.035303 17025697

[B104] Xia HM Wan SYM Shu C Chew YT Chaotic micromixers using two-layer crossing channels to exhibit fast mixing at low Reynolds numbers Lab on a Chip 2005 5 748 755 10.1039/b502031j 15970968

[B105] Cabral JT Hudson SD Microfluidic approach for rapid multicomponent interfacial tensiometry Lab on a Chip 2006 6 427 436 10.1039/b511976f 16511627

[B106] Frenz L Blank K Brouzes E Griffiths AD Reliable microfluidic on-chip incubation of droplets in delay-lines Lab on a Chip 2009 9 1344 1348 10.1039/b816049j 19417899 PMC5317046

[B107] Prakash M Gershenfeld N Microfluidic bubble logic Science 2007 315 832 835 10.1126/science.1136907 17289994

[B108] Boedicker JQ Li L Kline TR Ismagilov RF Detecting bacteria and determining their susceptibility to antibiotics by stochastic confinement in nanoliter droplets using plug-based microfluidics Lab on a Chip 2008 8 1265 1272 10.1039/b804911d 18651067 PMC2612531

[B109] Zeng Y Novak R Shuga J Smith MT Mathies RA High-Performance Single Cell Genetic Analysis Using Microfluidic Emulsion Generator Arrays Analytical Chemistry 2010 82 3183 3190 10.1021/ac902683t 20192178 PMC2859697

[B110] Mazutis L Araghi AF Miller OJ Baret JC Frenz L Janoshazi A Taly V Miller BJ Hutchison JB Link D Griffiths AD Ryckelnck M Droplet-Based Microfluidic Systems for High-Throughput Single DNA Molecule Isothermal Amplification and Analysis Analytical Chemistry 2009 81 4813 4821 10.1021/ac900403z 19518143

[B111] Brouzes E Medkova M Savenelli N Marran D Twardowski M Hutchison JB Rothberg JM Link DR Perrimon N Samuels ML Droplet microfluidic technology for single-cell high-throughput screening Proceedings of the National Academy of Sciences of the United States of America 2009 106 14195 14200 10.1073/pnas.0903542106 19617544 PMC2732882

[B112] Baret JC Miller OJ Taly V Ryckelynck M El-Harrak A Frenz L Rick C Samuels ML Hutchison JB Agresti JJ Link DR Weitz DA Griffiths AD Fluorescence-activated droplet sorting (FADS): efficient microfluidic cell sorting based on enzymatic activity Lab on a Chip 2009 9 1850 1858 10.1039/b902504a 19532959

[B113] Amemiya T Hashimoto K Fujishima A Faradaic Charge-Transfer with Double-Layer Charging and or Adsorption-Related Charging at Polymer-Modified Electrodes as Observed by Color Impedance Spectroscopy Journal of Physical Chemistry 1993 97 9736 9740 10.1021/j100140a033

[B114] Abdelgawad M Wheeler AR The Digital Revolution: A New Paradigm for Microfluidics Advanced Materials 2009 21 920 925 10.1002/adma.200802244

[B115] Wheeler AR Chemistry - Putting electrowetting to work Science 2008 322 539 540 10.1126/science.1165719 18948529

[B116] Abdelgawad M Park P Wheeler AR Optimization of device geometry in single-plate digital microfluidics Journal of Applied Physics 2009 105

[B117] Cho SK Moon HJ Kim CJ Creating, transporting, cutting, and merging liquid droplets by electrowetting-based actuation for digital microfluidic circuits Journal of Microelectromechanical Systems 2003 12 70 80 10.1109/JMEMS.2002.807467

[B118] Fouillet Y Jary D Chabrol C Claustre P Peponnet C Digital microfluidic design and optimization of classic and new fluidic functions for lab on a chip systems Microfluidics and Nanofluidics 2008 4 159 165 10.1007/s10404-007-0164-5

[B119] Gong J Kim CJ All-electronic droplet generation on-chip with real-time feedback control for EWOD digital microfluidics Lab on a Chip 2008 8 898 906 10.1039/b717417a 18497909 PMC2769494

[B120] Cooney CG Chen CY Emerling MR Nadim A Sterling JD Electrowetting droplet microfluidics on a single planar surface Microfluidics and Nanofluidics 2006 2 435 446 10.1007/s10404-006-0085-8

[B121] Yi UC Kim CJ Characterization of electrowetting actuation on addressable single-side coplanar electrodes J Micromech Microeng 2006 16 2053 2059 10.1088/0960-1317/16/10/018

[B122] Zhao YJ Cho SK Micro air bubble manipulation by electrowetting on dielectric (EWOD): transporting, splitting, merging and eliminating of bubbles Lab on a Chip 2007 7 273 280 10.1039/b616845k 17268631

[B123] Priest C Herminghaus S Seemann R Controlled electrocoalescence in microfluidics: Targeting a single lamella Applied Physics Letters 2006 89 134101 10.1063/1.2357039

[B124] Niu XZ Gielen F deMello AJ Edel JB Electro-Coalescence of Digitally Controlled Droplets Analytical Chemistry 2009 81 7321 7325 10.1021/ac901188n 19715363

[B125] Paik P Pamula VK Pollack MG Fair RB Electrowetting-based droplet mixers for microfluidic systems Lab on a Chip 2003 3 28 33 10.1039/b210825a 15100802

[B126] Paik P Pamula VK Fair RB Rapid droplet mixers for digital microfluidic systems Lab on a Chip 2003 3 253 259 10.1039/b307628h 15007455

[B127] Lu HW Bottausci F Fowler JD Bertozzi AL Meinhart C Kim CJ A study of EWOD-driven droplets by PIV investigation Lab on a Chip 2008 8 456 461 10.1039/b717141b 18305865 PMC2790058

[B128] Schertzer MJ Ben-Mrad R Sullivan PE Using capacitance measurements in EWOD devices to identify fluid composition and control droplet mixing Sensors and Actuators B-Chemical 2010 145 340 347 10.1016/j.snb.2009.12.019

[B129] Fan SK Huang PW Wang TT Peng YH Cross-scale electric manipulations of cells and droplets by frequency-modulated dielectrophoresis and electrowetting Lab on a Chip 2008 8 1325 1331 10.1039/b803204a 18651075

[B130] Cho SK Zhao YJ Kim CJ Concentration and binary separation of micro particles for droplet-based digital microfluidics Lab on a Chip 2007 7 490 498 10.1039/b615665g 17389966

[B131] Park SY Teitell MA Chiou EPY Single-sided continuous optoelectrowetting (SCOEW) for droplet manipulation with light patterns Lab on a Chip 2010 10 1655 1661 10.1039/c001324b 20448870

[B132] Chiou PY Park SY Wu MC Continuous optoelectrowetting for picoliter droplet manipulation Applied Physics Letters 2008 93 221110 10.1063/1.3039070

[B133] Chuang HS Kumar A Wereley ST Open optoelectrowetting droplet actuation Applied Physics Letters 2008 93 064104 10.1063/1.2970047

[B134] Chiou PY Chang ZH Wu MC Droplet manipulation with light on optoelectrowetting device Journal of Microelectromechanical Systems 2008 17 133 138

[B135] Luk VN Wheeler AR A Digital Microfluidic Approach to Proteomic Sample Processing Analytical Chemistry 2009 81 4524 4530 10.1021/ac900522a 19476392

[B136] Wheeler AR Moon H Bird CA Loo RRO Kim CJ Loo JA Garrell RL Digital microfluidics with in-line sample purification for proteomics analyses with MALDI-MS Analytical Chemistry 2005 77 534 540 10.1021/ac048754+ 15649050

[B137] Barbulovic-Nad I Au SH Wheeler AR A microfluidic platform for complete mammalian cell culture Lab on a Chip 2010 10 1536 1542 10.1039/c002147d 20393662

[B138] Shah GJ Ohta AT Chiou EPY Wu MC Kim CJ EWOD-driven droplet microfluidic device integrated with optoelectronic tweezers as an automated platform for cellular isolation and analysis Lab on a Chip 2009 9 1732 1739 10.1039/b821508a 19495457

[B139] Hua ZS Rouse JL Eckhardt AE Srinivasan V Pamula VK Schell WA Benton JL Mitchell TG Pollack MG Multiplexed Real-Time Polymerase Chain Reaction on a Digital Microfluidic Platform Analytical Chemistry 2010 82 2310 2316 10.1021/ac902510u 20151681 PMC2859674

[B140] Gong J Kim CJ Direct-referencing two-dimensional-array digital microfluidics using multilayer printed circuit board Journal of Microelectromechanical Systems 2008 17 257 264 19234613 10.1109/JMEMS.2007.912698 PMC2645069

[B141] Luk VN Mo GCH Wheeler AR Pluronic additives: A solution to sticky problems in digital microfluidics Langmuir 2008 24 6382 6389 10.1021/la7039509 18481875

[B142] Yang H Luk VN Abeigawad M Barbulovic-Nad I Wheeer AR A World-to-Chip Interface for Digital Microfluidics Analytical Chemistry 2009 81 1061 1067 10.1021/ac802154h 19115860

[B143] Bousse L Cohen C Nikiforov T Chow A Kopf-Sill AR Dubrow R Parce JW Electrokinetically controlled microfluidic analysis systems ANNU REV BIOPH BIOM 2000 29 155 181 10.1146/annurev.biophys.29.1.155 10940246

[B144] Ramos A Morgan H Green NG Castellanos A Ac electrokinetics: a review of forces in microelectrode structures Journal of Physics D-Applied Physics 1998 31 2338 2353 10.1088/0022-3727/31/18/021

[B145] Sin MLY Gau V Liao JC Haake DA Wong PK Active Manipulation of Quantum Dots using AC Electrokinetics J Phys Chem C 2009 113 6561 6565 10.1021/jp9004423

[B146] Sin MLY Shimabukuro Y Wong PK Hybrid electrokinetics for separation, mixing, and concentration of colloidal particles Nanotechnology 2009 20 165701 10.1088/0957-4484/20/16/165701 19420574

[B147] Ghosal S Fluid mechanics of electroosmotic flow and its effect on band broadening in capillary electrophoresis Electrophoresis 2004 25 214 228 10.1002/elps.200305745 14743475

[B148] Ermolina I Morgan H The electrokinetic properties of latex particles: comparison of electrophoresis and dielectrophoresis J Colloid Interf Sci 2005 285 419 428 10.1016/j.jcis.2004.11.003 15797441

[B149] Chen L Lee S Choo J Lee EK Continuous dynamic flow micropumps for microfluid manipulation J Micromech Microeng 2008 18 013001 10.1088/0960-1317/18/1/013001

[B150] Mruetusatorn P Mahfouz MR Wu J Low-voltage dynamic control for DC electroosmotic devices Sensor Actuat a-Phys 2009 153 237 243 10.1016/j.sna.2009.05.011

[B151] Unni HN Keh HJ Yang C Analysis of electrokinetic transport of a spherical particle in a microchannel Electrophoresis 2007 28 658 664 10.1002/elps.200600576 17304499

[B152] Castellanos A Ramos A Gonzalez A Green NG Morgan H Electrohydrodynamics and dielectrophoresis in microsystems: scaling laws Journal of Physics D-Applied Physics 2003 36 2584 2597 10.1088/0022-3727/36/20/023

[B153] Visser KR Electric-Conductivity of Stationary and Flowing Human Blood at Low-Frequencies Medical & Biological Engineering & Computing 1992 30 636 640 1297019 10.1007/BF02446796

[B154] Li FA Huang JL Shen SY Wang CW Her GR Development of a Liquid-Junction/Low-Flow Interface for Phosphate Buffer Capillary Electrophoresis Mass Spectrometry Analytical Chemistry 2009 81 2810 2814 10.1021/ac802491y 19245229

[B155] Breadmore MC Quirino JP 100 000-fold concentration of anions in capillary zone electrophoresis using electroosmotic flow controlled counterflow isotachophoretic stacking under field amplified conditions Analytical Chemistry 2008 80 6373 6381 10.1021/ac8007835 18627177

[B156] Lin CH Wang JH Fu LM Improving the separation efficiency of DNA biosamples in capillary electrophoresis microchips using high-voltage pulsed DC electric fields Microfluidics and Nanofluidics 2008 5 403 410 10.1007/s10404-008-0259-7

[B157] Bertilsson S Cavanaugh CM Polz MF Sequencing-independent method to generate oligonucleotide probes targeting a variable region in bacterial 16S rRNA by PCR with detachable primers Applied and Environmental Microbiology 2002 68 6077 6086 10.1128/AEM.68.12.6077-6086.2002 12450831 PMC134391

[B158] Lacher NA Roberts RK He Y Cargill H Kearns KM Holovics H Ruesch MN Development, validation, and implementation of capillary gel electrophoresis as a replacement for SDS-PAGE for purity analysis of IgG2 mAbs Journal of Separation Science 2010 33 218 227 10.1002/jssc.200900597 20087870

[B159] Lacher NA Wang O Roberts RK Holovics HJ Aykent S Schlittler MR Thompson MR Demarest CW Development of a capillary gel electrophoresis method for monitoring disulfide isomer heterogeneity in IgG2 antibodies Electrophoresis 2010 31 448 458 10.1002/elps.200900371 20119952

[B160] Swanson P Gelbart R Atlas E Yang L Grogan T Butler WF Ackley DE Sheldon E A fully multiplexed CMOS biochip for DNA analysis Sensors and Actuators B-Chemical 2000 64 22 30 10.1016/S0925-4005(99)00478-5

[B161] Sosnowski RG Tu E Butler WF OConnell JP Heller MJ Rapid determination of single base mismatch mutations in DNA hybrids by direct electric field control Proceedings of the National Academy of Sciences of the United States of America 1997 94 1119 1123 10.1073/pnas.94.4.1119 9037016 PMC19754

[B162] Gao J Yin XF Fang ZL Integration of single cell injection, cell lysis, separation and detection of intracellular constituents on a microfluidic chip Lab on a Chip 2004 4 47 52 10.1039/b310552k 15007440

[B163] Lee DW Cho YH A continuous electrical cell lysis device using a low dc voltage for a cell transport and rupture Sensors and Actuators B-Chemical 2007 124 84 89 10.1016/j.snb.2006.11.054

[B164] Chang CC Yang RJ Electrokinetic mixing in microfluidic systems Microfluidics and Nanofluidics 2007 3 501 525 10.1007/s10404-007-0178-z

[B165] Ng WY Goh S Lam YC Yang C Rodriguez I DC-biased AC-electroosmotic and AC-electrothermal flow mixing in microchannels Lab on a Chip 2009 9 802 809 10.1039/b813639d 19255662

[B166] Du E Manoochehri S Microfluidic pumping optimization in microgrooved channels with ac electrothermal actuations Applied Physics Letters 2010 96 034102 10.1063/1.3280076

[B167] Gagnon ZR Chang HC Electrothermal ac electro-osmosis Applied Physics Letters 2009 94 024101 10.1063/1.3020720

[B168] Wang XY Cheng C Wang SL Liu SR Electroosmotic pumps and their applications in microfluidic systems Microfluidics and Nanofluidics 2009 6 145 162 10.1007/s10404-008-0399-9 20126306 PMC2756694

[B169] Williams SJ Kumar A Green NG Wereley ST Optically induced electrokinetic concentration and sorting of colloids J Micromech Microeng 2010 20 015022 10.1088/0960-1317/20/1/015022

[B170] Lei KF Cheng H Choy KY Chow LMC Electrokinetic DNA concentration in microsystems Sensor Actuat a-Phys 2009 156 381 387 10.1016/j.sna.2009.10.006

[B171] Zhu K Kaprelyants AS Salina EG Markx GH Separation by dielectrophoresis of dormant and nondormant bacterial cells of Mycobacterium smegmatis Biomicrofluidics 2010 4 022809 10.1063/1.3435335 20697591 PMC2917864

[B172] Huber DE Markel ML Pennathur S Patel KD Oligonucleotide hybridization and free-solution electrokinetic separation in a nanofluidic device Lab on a Chip 2009 9 2933 2940 10.1039/b901739a 19789746

[B173] Pommer MS Zhang YT Keerthi N Chen D Thomson JA Meinhart CD Soh HT Dielectrophoretic separation of platelets from diluted whole blood in microfluidic channels Electrophoresis 2008 29 1213 1218 10.1002/elps.200700607 18288670

[B174] Krishnan R Sullivan BD Mifflin RL Esener SC Heller MJ Alternating current electrokinetic separation and detection of DNA nanoparticles in high-conductance solutions Electrophoresis 2008 29 1765 1774 10.1002/elps.200800037 18393345

[B175] Wong PK Chen CY Wang TH Ho CM Electrokinetic bioprocessor for concentrating cells and molecules Analytical Chemistry 2004 76 6908 6914 10.1021/ac049479u 15571340

[B176] Lvovich VF Matthews E Riga AT Kaza L AC electrokinetic platform for iontophoretic transdermal drug delivery Journal of Controlled Release 2010 145 134 140 10.1016/j.jconrel.2010.04.015 20420867

[B177] Gagnon Z Mazur J Chang HC Integrated AC electrokinetic cell separation in a closed-loop device Lab on a Chip 2010 10 718 726 10.1039/b917220c 20221559

[B178] Sin MLY Gau V Liao JC Wong PK Electrothermal Fluid Manipulation of High-Conductivity Samples for Laboratory Automation Applications Jala-J Assoc Lab Aut 2010 15 426 432 10.1016/j.jala.2010.05.004 PMC3003926 21180401

[B179] Gorkin R Park J Siegrist J Amasia M Lee BS Park JM Kim J Kim H Madou M Cho YK Centrifugal microfluidics for biomedical applications Lab on a Chip 2010 10 1758 1773 10.1039/b924109d 20512178

[B180] Duffy DC Gillis HL Lin J Sheppard NF Kellogg GJ Microfabricated centrifugal microfluidic systems: Characterization and multiple enzymatic assays Analytical Chemistry 1999 71 4669 4678 10.1021/ac990682c

[B181] Madou M Zoval J Jia GY Kido H Kim J Kim N Lab on a CD Annual Review of Biomedical Engineering 2006 8 601 628 10.1146/annurev.bioeng.8.061505.095758 16834568

[B182] Chen JM Huang PC Lin MG Analysis and experiment of capillary valves for microfluidics on a rotating disk Microfluidics and Nanofluidics 2008 4 427 437 10.1007/s10404-007-0196-x

[B183] Siegrist J Gorkin R Clime L Roy E Peytavi R Kido H Bergeron M Veres T Madou M Serial siphon valving for centrifugal microfluidic platforms Microfluidics and Nanofluidics 2010 9 55 63 10.1007/s10404-009-0523-5

[B184] Park JM Cho YK Lee BS Lee JG Ko C Multifunctional microvalves control by optical illumination on nanoheaters and its application in centrifugal microfluidic devices Lab on a Chip 2007 7 557 564 10.1039/b616112j 17476373

[B185] Mark D Metz T Haeberle S Lutz S Ducree J Zengerle R von Stetten F Centrifugo-pneumatic valve for metering of highly wetting liquids on centrifugal microfluidic platforms Lab on a Chip 2009 9 3599 3603 10.1039/b914415c 20024042

[B186] Grumann M Geipel A Riegger L Zengerle R Ducree J Batch-mode mixing on centrifugal microfluidic platforms Lab on a Chip 2005 5 560 565 10.1039/b418253g 15856095

[B187] Lutz VR S Mark D Ducree J Zengerle R von Stetten F Unidirectional Shake-Mode for Mixing Highly Wetting Fluids on Centrifugal Platforms Twelfth International Conference on Miniaturized Systems for Chemistry and Life Sciences; 12-16 October; 2008 San Diego, California, USA 748 750

[B188] Noroozi Z Kido H Micic M Pan HS Bartolome C Princevac M Zoval J Madou M Reciprocating flow-based centrifugal microfluidics mixer Review of Scientific Instruments 2009 80 075102 10.1063/1.3169508 19655976

[B189] Haeberle S Brenner T Zengerle R Ducree J Centrifugal extraction of plasma from whole blood on a rotating disk Lab on a Chip 2006 6 776 781 10.1039/b604145k 16738730

[B190] Siegrist J Gorkin R Bastien M Stewart G Peytavi R Kido H Bergeron M Madou M Validation of a centrifugal microfluidic sample lysis and homogenization platform for nucleic acid extraction with clinical samples Lab on a Chip 2010 10 363 371 10.1039/b913219h 20091009

[B191] Martinez-Duarte R Gorkin RA Abi-Samra K Madou MJ The integration of 3D carbon-electrode dielectrophoresis on a CD-like centrifugal microfluidic platform Lab on a Chip 2010 10 1030 1043 10.1039/b925456k 20358111

[B192] Focke M Stumpf F Faltin B Reith P Bamarni D Wadle S Muller C Reinecke H Schrenzel J Francois P Mark D Roth G Zengerle R von Stetten F Microstructuring of polymer films for sensitive genotyping by real-time PCR on a centrifugal microfluidic platform Lab on a Chip 2010 10 2519 2526 10.1039/c004954a 20607174

[B193] Lutz S Weber P Focke M Faltin B Hoffmann J Muller C Mark D Roth G Munday P Armes N Piepenburg O Zengerle R von Stetten F Microfluidic lab-on-a-foil for nucleic acid analysis based on isothermal recombinase polymerase amplification (RPA) Lab on a Chip 2010 10 887 893 10.1039/b921140c 20300675

[B194] Lee BS Lee JN Park JM Lee JG Kim S Cho YK Ko C A fully automated immunoassay from whole blood on a disc Lab on a Chip 2009 9 1548 1555 10.1039/b820321k 19458861

[B195] Lafleur JP Rackov AA McAuley S Salin ED Miniaturised centrifugal solid phase extraction platforms for in-field sampling, pre-concentration and spectrometric detection of organic pollutants in aqueous samples Talanta 2010 81 722 726 10.1016/j.talanta.2009.12.001 20188988

[B196] Dubertret B Calame M Libchaber AJ Single-mismatch detection using gold-quenched fluorescent oligonucleotides (vol 19, pg 365, 2001) Nature Biotechnology 2001 19 680 681 10.1038/86762 11283596

[B197] Duffy DC McDonald JC Schueller OJA Whitesides GM Rapid prototyping of microfluidic systems in poly(dimethylsiloxane) Analytical Chemistry 1998 70 4974 4984 10.1021/ac980656z 21644679

[B198] Watson MWL Jebrail MJ Wheeler AR Multilayer Hybrid Microfluidics: A Digital-to-Channel Interface for Sample Processing and Separations Analytical Chemistry 2010 82 6680 6686 10.1021/ac101379g 20670000

[B199] Smith JE Medley CD Tang ZW Shangguan D Lofton C Tan WH Aptamer-conjugated nanoparticles for the collection and detection of multiple cancer cells Analytical Chemistry 2007 79 3075 3082 10.1021/ac062151b 17348633

[B200] Monaghan PB McCarney KM Ricketts A Littleford RE Docherty F Smith WE Graham D Cooper JM Bead-based DNA diagnostic assay for chlamydia using nanoparticle-mediated surface-enhanced resonance Raman scattering detection within a lab-on-a-chip format Analytical Chemistry 2007 79 2844 2849 10.1021/ac061769i 17326610

[B201] Krishnamoorthy G Carlen ET Bomer JG Wijnperle D deBoer HL van den Berg A Schasfoort RBM Electrokinetic label-free screening chip: a marriage of multiplexing and high throughput analysis using surface plasmon resonance imaging Lab on a Chip 2010 10 986 990 10.1039/c000705f 20358104

[B202] Qian XM Peng XH Ansari DO Yin-Goen Q Chen GZ Shin DM Yang L Young AN Wang MD Nie SM In vivo tumor targeting and spectroscopic detection with surface-enhanced Raman nanoparticle tags Nature Biotechnology 2008 26 83 90 10.1038/nbt1377 18157119

[B203] Moore BD Stevenson L Watt A Flitsch S Turner NJ Cassidy C Graham D Rapid and ultra-sensitive determination of enzyme activities using surface-enhanced resonance Raman scattering Nature Biotechnology 2004 22 1133 1138 10.1038/nbt1003 15300259

[B204] Novak L Neuzil P Pipper J Zhang Y Lee SH An integrated fluorescence detection system for lab-on-a-chip applications Lab on a Chip 2007 7 27 29 10.1039/b611745g 17180202

[B205] Seo S Isikman SO Sencan I Mudanyali O Su TW Bishara W Erlinger A Ozcan A High-Throughput Lens-Free Blood Analysis on a Chip Analytical Chemistry 2010 82 4621 4627 10.1021/ac1007915 20450181 PMC2892055

[B206] Coskun AF Su TW Ozcan A Wide field-of-view lens-free fluorescent imaging on a chip Lab on a Chip 2010 10 824 827 10.1039/b926561a 20379564 PMC2863091

[B207] Su TW Erlinger A Tseng D Ozcan A Compact and Light-Weight Automated Semen Analysis Platform Using Lensfree on-Chip Microscopy Analytical Chemistry 2010 82 8307 8312 10.1021/ac101845q 20836503 PMC2987715

[B208] Zheng GA Lee SA Yang S Yang CH Sub-pixel resolving optofluidic microscope for on-chip cell imaging Lab on a Chip 2010 10 3125 3129 10.1039/c0lc00213e 20877904

[B209] Wu JG Cui XQ Zheng GA Wang YM Lee LM Yang CH Wide field-of-view microscope based on holographic focus grid illumination Opt Lett 2010 35 2188 2190 10.1364/OL.35.002188 20596189

[B210] Tseng D Mudanyali O Oztoprak C Isikman SO Sencan I Yaglidere O Ozcan A Lensfree microscopy on a cellphone Lab on a Chip 2010 10 1787 1792 10.1039/c003477k 20445943 PMC2941438

[B211] Wang J Electrochemical biosensors: Towards point-of-care cancer diagnostics Biosensors & Bioelectronics 2006 21 1887 1892 10.1016/j.bios.2005.10.027 16330202

[B212] Dungchai W Chailapakul O Henry CS Electrochemical Detection for Paper-Based Microfluidics Analytical Chemistry 2009 81 5821 5826 10.1021/ac9007573 19485415

[B213] Ricci F Adornetto G Moscone D Plaxco KW Palleschi G Quantitative, reagentless, single-step electrochemical detection of anti-DNA antibodies directly in blood serum Chem Commun 2010 46 1742 1744 10.1039/b922595a PMC3082467 20177635

[B214] Blow N Microfluidics: in search of a killer application Nat Methods 2007 4 665 668 10.1038/nmeth0807-665

[B215] Chung S Sudo R Vickerman V Zervantonakis IK Kamm RD Microfluidic Platforms for Studies of Angiogenesis, Cell Migration, and Cell-Cell Interactions Annals of Biomedical Engineering 2010 38 1164 1177 10.1007/s10439-010-9899-3 20336839

[B216] Zare RN Kim S Microfluidic Platforms for Single-Cell Analysis Annual Review of Biomedical Engineering, Vol 12 2010 12 187 201 10.1146/annurev-bioeng-070909-105238 20433347

[B217] Gunther A Yasotharan S Vagaon A Lochovsky C Pinto S Yang JL Lau C Voigtlaender-Bolz J Bolz SS A microfluidic platform for probing small artery structure and function Lab on a Chip 2010 10 2341 2349 10.1039/c004675b 20603685 PMC3753293

[B218] Li G Chen Q Li JJ Hu XJ Zhao JL A Compact Disk-Like Centrifugal Microfluidic System for High-Throughput Nanoliter-Scale Protein Crystallization Screening Analytical Chemistry 2010 82 4362 4369 10.1021/ac902904m 20459060

[B219] Huang XH Gordon MJ Zare RN Current-Monitoring Method for Measuring the Electroosmotic Flow-Rate in Capillary Zone Electrophoresis Analytical Chemistry 1988 60 1837 1838 10.1021/ac00168a040

[B220] Joanicot M Ajdari A Applied physics - Droplet control for microfluidics Science 2005 309 887 888 10.1126/science.1112615 16081724

[B221] Bringer MR Gerdts CJ Song H Tice JD Ismagilov RF Microfluidic systems for chemical kinetics that rely on chaotic mixing in droplets Philos T Roy Soc A 2004 362 1087 1104 10.1098/rsta.2003.1364 PMC1769314 15306486

[B222] Pollack MG Shenderov AD Fair RB Electrowetting-based actuation of droplets for integrated microfluidics Lab on a Chip 2002 2 96 101 10.1039/b110474h 15100841

